# *Ralstonia solanacearum* promotes pathogenicity by utilizing l‐glutamic acid from host plants

**DOI:** 10.1111/mpp.12963

**Published:** 2020-06-29

**Authors:** Fangfang Shen, Wenfang Yin, Shihao Song, Zhihan Zhang, Peiyi Ye, Yong Zhang, Jianuan Zhou, Fei He, Peng Li, Yinyue Deng

**Affiliations:** ^1^ College of Agriculture South China Agricultural University Guangzhou China; ^2^ College of Resources and Environment Southwest University Chongqing China; ^3^ Ministry of Education Key Laboratory for Ecology of Tropical Islands, Key Laboratory of Tropical Animal and Plant Ecology of Hainan Province College of Life Sciences Hainan Normal University Haikou China; ^4^ School of Pharmaceutical Sciences (Shenzhen) Sun Yat‐sen University Guangzhou China

**Keywords:** l‐glutamic acid, pathogen–host interaction, *Ralstonia solanacearum*, virulence

## Abstract

*Ralstonia solanacearum* is an important bacterial pathogen that can infect a broad range of plants worldwide. A previous study showed that *R. solanacearum* could respond to exogenous organic acids or amino acids to modulate cell motility. However, it was unclear whether *R. solanacearum* uses these compounds to control infection. In this study, we found that *R. solanacearum* GMI1000 uses host plant metabolites to enhance the biosynthesis of virulence factors. We demonstrated that l‐glutamic acid from host plants is the key active component associated with increased extracellular polysaccharide production, cellulase activity, swimming motility, and biofilm formation in *R. solanacearum* GMI1000. In addition, l‐glutamic acid also promoted colonization of *R. solanacearum* cells in the roots and stems of tomato plants and accelerated disease incidence. Furthermore, genetic screening and biochemical analysis suggested that *RS01577*, a hybrid sensor histidine kinase/response regulator, is involved in l‐glutamic acid signalling in *R. solanacearum*. Mutations in *RS01577* and exogenous addition of l‐glutamic acid to the GMI1000 wild‐type strain had overlapping effects on both the transcriptome and biological functions of *R. solanacearum*, including on motility, biofilm formation, and virulence. Thus, our results have established a new interaction mechanism between *R. solanacearum* and host plants that highlights the complexity of the virulence regulation mechanism and may provide new insight into disease control.

## Graphical abstract

*Ralstonia solanacearum* perceives l‐glutamic acid from host plants to enhance extracellular polysaccharide production, cellulase activity, swimming motility, biofilm formation, and virulence.

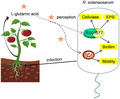

## 1 INTRODUCTION

*Ralstonia solanacearum* is a notorious soilborne bacterium that can cause disastrous bacterial wilt in hundreds of plantspecies, including important crops such as tomato, potato, banana, eggplant, and pepper (Hayward, 1991). *R. solanacearum* is considered to exist as a complex that consists of many genetically distinct strains, commonly called the *R. solanacearum* species complex (RSSC; Allen *et al*., 2006). *R. solanacearum* can produce multiple virulence factors during the infection process, resulting in typical wilting symptoms in host plants. Numerous studies have shown that extracellular polysaccharides (EPS) and cell wall‐degrading enzymes (CWDE) are the major virulence factors of *R. solanacearum* responsible for symptoms of wilt (Genin and Denny, 2012). Decreased EPS production and extracellular enzyme activities lead to reduced virulence (Huang and Allen, 1997; Milling *et al*., 2011). In addition, motility and biofilm formation also contribute to the colonization and infection behaviours of *R. solanacearum* (Tans‐Kersten *et al*., 2001; Yao and Allen, 2007).

Many studies have revealed that *R. solanacearum* can manipulate compounds from host plant cells. For example, galacturonic acid released by extracellular polygalacturonases from plant cell walls can be used to nourish bacterial pathogen cells and accelerate the disease progression of bacterial wilt (Huang and Allen, 2000; Gonzalez and Allen, 2003). *R. solanacearum* was also shown to degrade plant salicylic acid (SA) to suppress host immunity and protect itself in plant hosts that use SA as a defence signalling molecule (Lowe‐Power *et al*., 2016). A variety of other organic substrates, such as tryptophan and methionine, can also be used by *R. solanacearum* to enhance virulence (Brown and Allen, 2004; Genin and Boucher, 2004; Plener *et al*., 2012). In addition, *R. solanacearum* uses exogenous organic acids and amino acids to modulate cell motility (Li *et al*., 2017).

l‐glutamic acid plays an important role in nutrient metabolism, energy supply, immune response, oxidative stress, and signal regulation (Brosnan and Brosnan, 2013). Notably, the loss of glutamate dehydrogenase in *R. solanacearum* resulted in reduced EPS production and bacterial virulence (Wu *et al*., 2015), demonstrating that glutamate dehydrogenase plays a vital role in pathogenicity. In plants, glutamate is absorbed by surrounding tissues and transferred to the protein synthesis centre through xylem vessels (Pratelli and Pilot, 2014). It can cause specific changes in growth, root tip morphology, and root branching (Price *et al*., 2012; Forde, 2014). Glutamate metabolism is also important for crucial metabolic functions associated with the plant defence against pathogens. Intriguingly, pathogens have evolved strategies to utilize amino acids from hosts to their own benefit. In this study, we first revealed that *R. solanacearum* utilized plant‐derived l‐glutamic acid to promote the production of multiple virulence factors and pathogenicity. We also demonstrated that *RS01577*, which is a hybrid sensor histidine kinase/response regulator, is involved in l‐glutamic acid signalling. Our results not only provide new insight into the interaction between host plants and *R. solanacearum*, but also reveal the effect of exogenous l‐glutamic acid on the pathogenicity of *R. solanacearum*.

## 2 RESULTS

### 2.1 Tomato extract induces EPS production and cellulase activity in *R. solanacearum*

**Figure 1:**
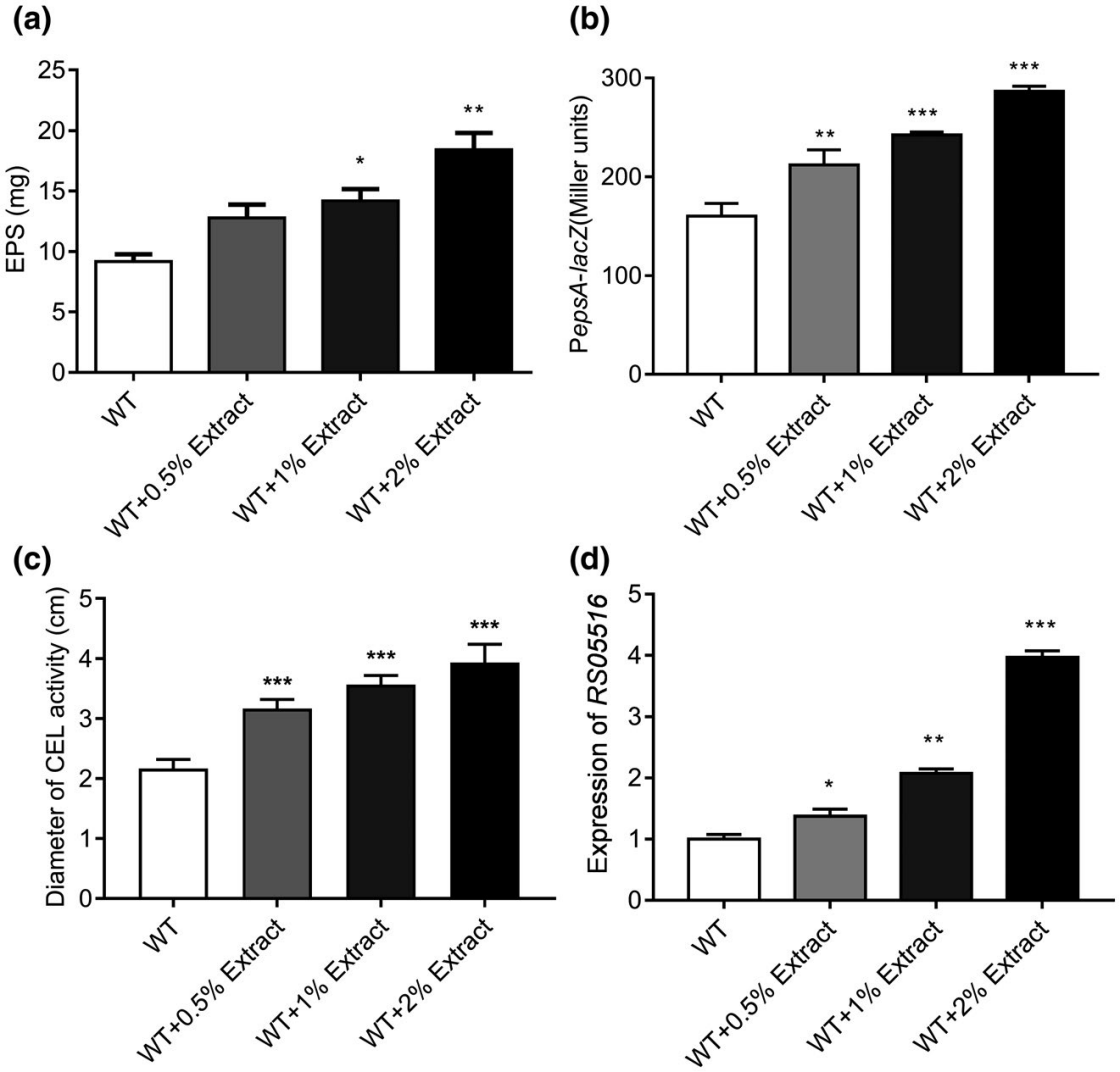
Tomato extract induces extracellular polysaccharide (EPS) production and cellulase activity in *Ralstonia solanacearum*. Effects of tomato extract on wild‐type (WT) *R. solanacearum* EPS production (a), the expression levels of the EPS‐related gene *epsA* (b), cellulase activity (c), and the cellulase‐encoding gene (d). EPS production and cellulase activity were induced by adding 0.5%, 1%, and 2% of tomato extract. Expression of the cellulase activity‐related gene *RS05516* was measured by quantitative reverse transcription PCR in CPG medium supplemented with different concentrations of tomato extract, whereas expression of *epsA* was measured based on β‐galactosidase activity using P*epsA*‐*LacZ* transcriptional fusions in various concentrations of tomato extract. The data are means ± *SD* of three independent experiments (unpaired *t* test, compared with the wild‐type strain GMI1000, *p < .05; **p < .01; ***p < .001)

To study whether the metabolic products of host plants influence virulence in *R. solanacearum*, we analysed the effect of tomato extract on the production of the virulence factors EPS and cellulase. The results show that exogenous addition of tomato extract notably increased EPS production (Figure 1a) but only slightly increased the bacterial growth rate (Figure S1). To test whether the tomato extract affected *epsA* (an EPS‐related gene) transcription, we used an *epsA* promoter‐*lacZ* fusion and measured the promoter activity in *R. solanacearum* GMI1000. Consistent with the above result, when the wild‐type strain was grown in the presence of tomato extract, *epsA* expression was induced by the tomato extract in a dose‐dependent manner (Figure 1b).

We next sought to measure cellulase activity in the wild‐type strain in the presence or absence of tomato extract. Our results revealed a notable induction of cellulase production in the wild‐type strain on treatment with tomato extract (Figure 1c). Then, the expression of the cellulase synthase‐encoding gene (*RS05516*) was also assessed by quantitative reverse transcription PCR (RT‐qPCR). The results demonstrate that the gene expression levels of *RS05516* increased upon supplementation with different amounts of tomato extract (Figure 1d).

### 2.2 Tomato extract enhances the motility activity and biofilm formation of *R. solanacearum*

**Figure 2:**
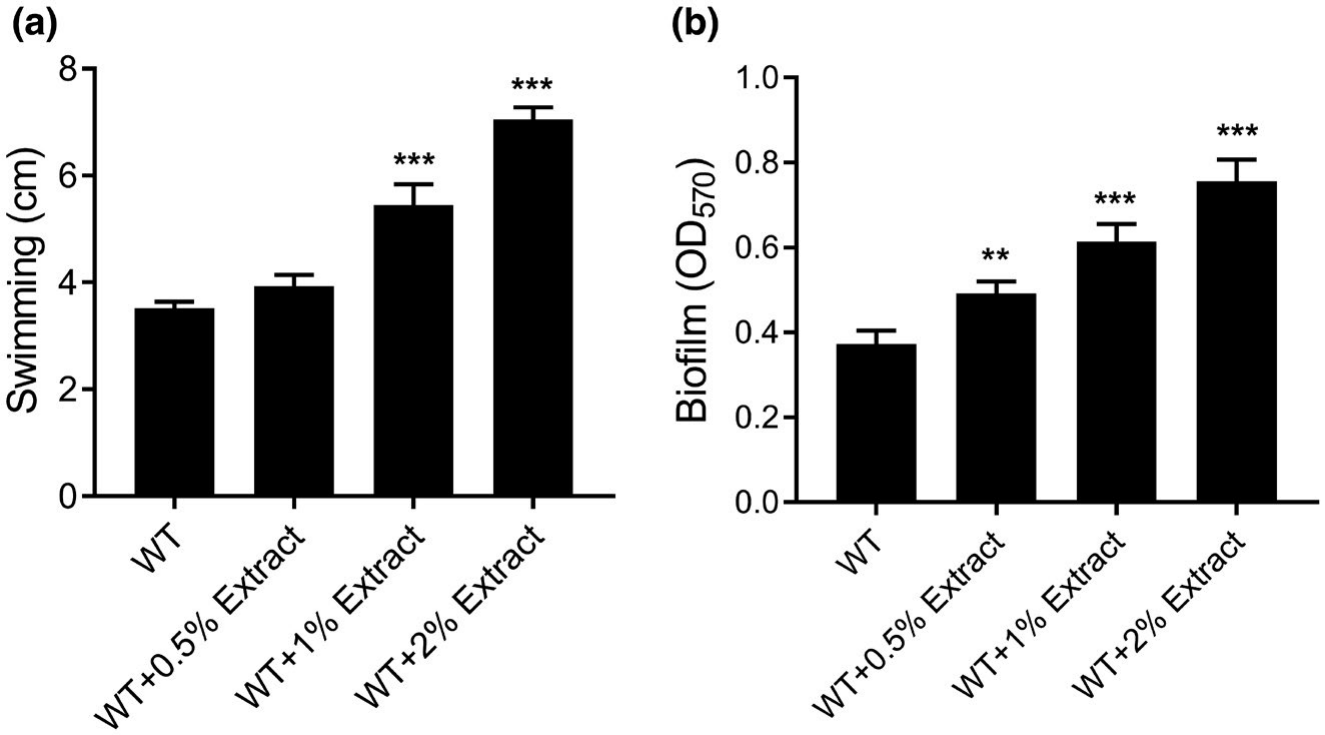
Tomato extract enhances the motility and biofilm formation activity of *Ralstonia solanacearum*. Effects of tomato extract on swimming motility (a) and biofilm formation (b). The data are means ± *SD* of three independent experiments (unpaired *t* test, compared with the wild‐type (WT) strain GMI1000, **p < .01; ***p < .001)

To further determine whether other virulence factors of *R. solanacearum* are also affected by the tomato extract, motility and biofilm formation were tested in the presence and absence of different concentrations of tomato extract. The addition of 0.5%, 1%, and 2% of tomato extract increased the swimming motility of GMI1000 to 1.12‐, 1.55‐, and 2.00‐fold of that observed for the untreated wild‐type strain (Figure 2a). Similarly, exogenous addition of tomato extract also significantly induced biofilm formation by *R. solanacearum* (Figure 2b).

### 2.3 l‐glutamic acid is an active component of tomato extract

**Figure 3:**
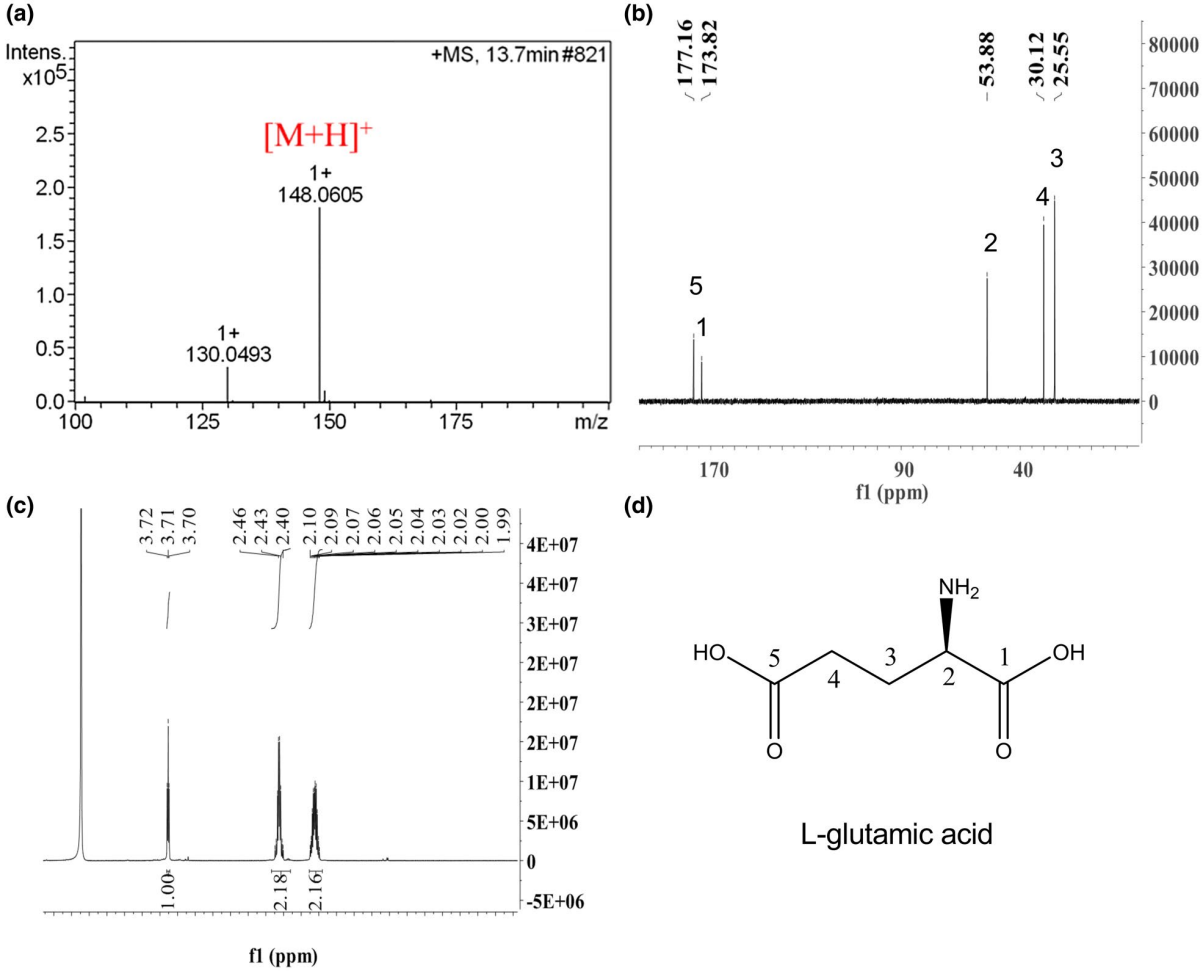
Structural characterization of l‐glutamic acid from the tomato extract to induce *Ralstonia solanacearum*: (a) Electrospray ionization mass spectrometry (ESI‐MS) spectrum of the compound, (b) ^13^C NMR spectrum of the compound, (c) ^1^H nuclear magnetic resonance (NMR) spectrum of the compound, and (d) structure of the compound

To identify the active component that induces EPS production, cellulase activity, motility, and biofilm formation in *R. solanacearum* GMI1000, we first isolated and purified the active fractions of tomato extract to induce cellulase activity of *R. solanacearum* GMI1000 using high performance liquid chromatography (HPLC) (Figure S2). Approximately 10 mg of purified compound with induction activity was obtained. High‐resolution electrospray ionization mass spectrometry (HR‐ESI‐MS) analysis of the active compound revealed a molecular ion [M + H]^+^ with an *m*/*z* ratio of 148.0605, consistent with a molecular formula of C_5_H_9_NO_4_ (Figure 3a). The ^13^C nuclear magnetic resonance (NMR) spectrum indicated the presence of two methylene groups (δC 30.1, 25.6), one methine group (δC 53.9), and two carbonyl groups (δC 177.2, 173.8; Figure 3b). The ^1^H NMR spectrum showed two methylenes at δH 2.46 (m, 2H) and δH 2.10 (m, 2H), and one methine at δH 3.72 (t, J = 6.3, 6.3 Hz; Figure 3c). These spectra are the same as those in the previous report (Ye *et al*., 1993). Thus, the active compound was determined to be l‐glutamic acid (Figure 3d).

### 2.4 l‐glutamic acid induces the production of virulence factors in *R. solanacearum*

**Figure 4:**
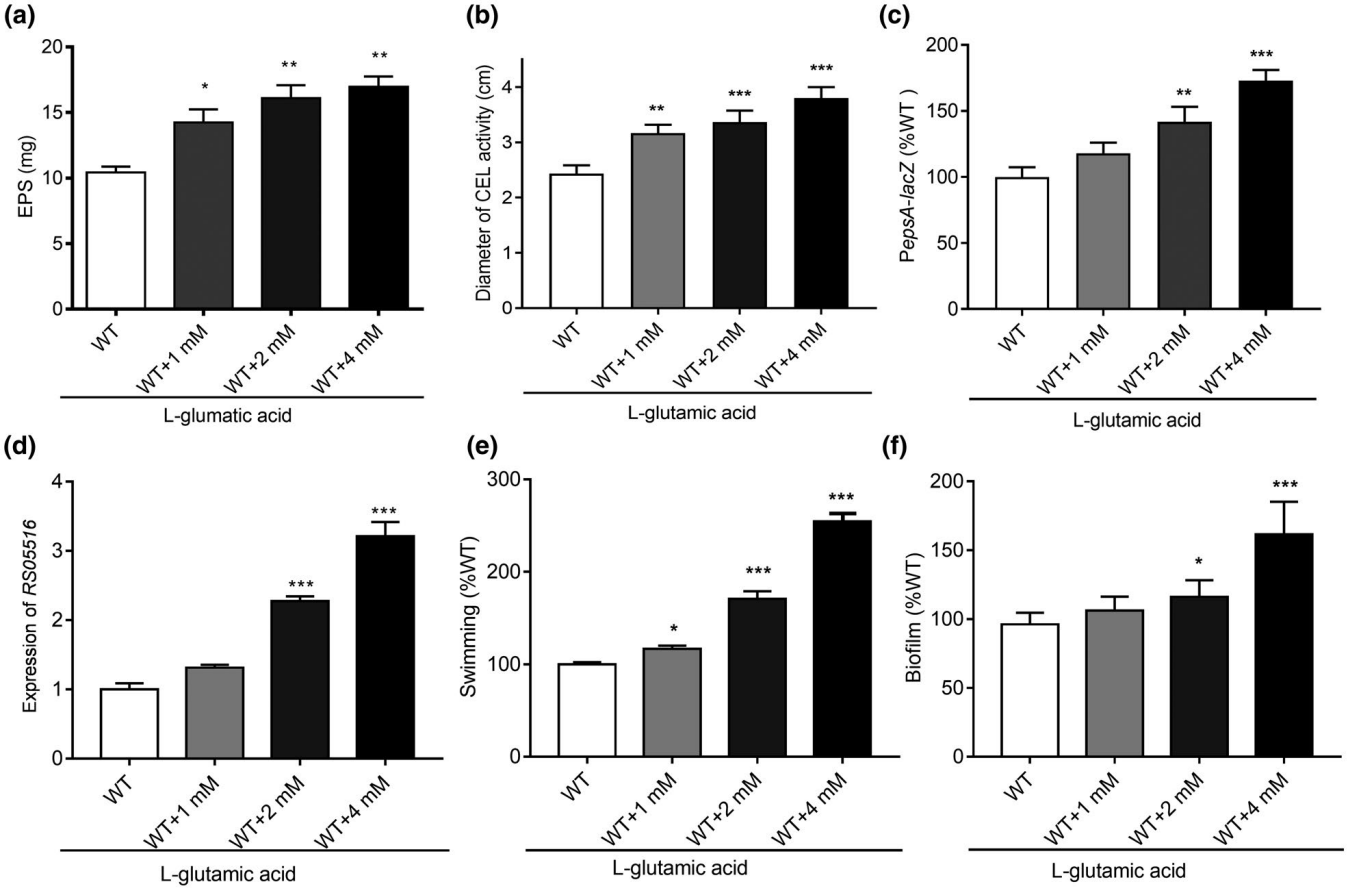
l‐glutamic acid induces virulence factor production in *Ralstonia solanacearum*. Effects of l‐glutamic acid on extracellular poysaccharide (EPS) production and cellulase activity in *R. solanacearum*. EPS production (a) and cellulase activity (b) were induced by adding different concentrations of l‐glutamic acid. (c) Expression of *epsA* was measured based on β‐galactosidase activity using P*epsA*‐*LacZ* transcriptional fusions with addition of different concentrations of l‐glutamic acid. (d) Expression of the cellulase activity‐related gene *RS05516* was measured by quantitative reverse transcription PCR in CPG medium supplemented with different concentrations of l‐glutamic acid. Effects of exogenous addition of different concentrations of l‐glutamic acid on motility (e) and biofilm formation (f). The data are means ± *SD* of three independent experiments (unpaired *t* test, compared with the wild‐type (WT) strain GMI1000, *p < .05; **p < .01; ***p < .001)

As l‐glutamic acid is one major active component in tomato extract that induces virulence factor production in *R. solanacearum* GMI1000 (Figures 1 and 2), we calculated the concentration of l‐glutamic acid in the extract using an automated amino acid analyser (Figure S3). The concentration of l‐glutamic acid in the stock extract was approximately 41.6 mM, and the final concentration of l‐glutamic acid in the bacterial culture supplemented with 0.5%, 1.0%, and 2.0% of the extract was therefore approximately 0.2, 0.4, and 0.8 mM, respectively. Consistent with the findings described above, we found that exogenous addition of l‐glutamic acid significantly enhanced the production of EPS and cellulase, and increased the expression of the corresponding genes in a dose‐dependent manner but did not affect the bacterial growth rate (Figures 4 and S4). In particular, addition of 4 mM l‐glutamic acid considerably enhanced the motility and biofilm formation of *R. solanacearum* GMI1000 by 157% and 52%, respectively (Figure 4e,f).

### 2.5 A hybrid sensor histidine kinase/response regulator is involved in intraspecies signalling of l‐glutamic acid

**Figure 5:**
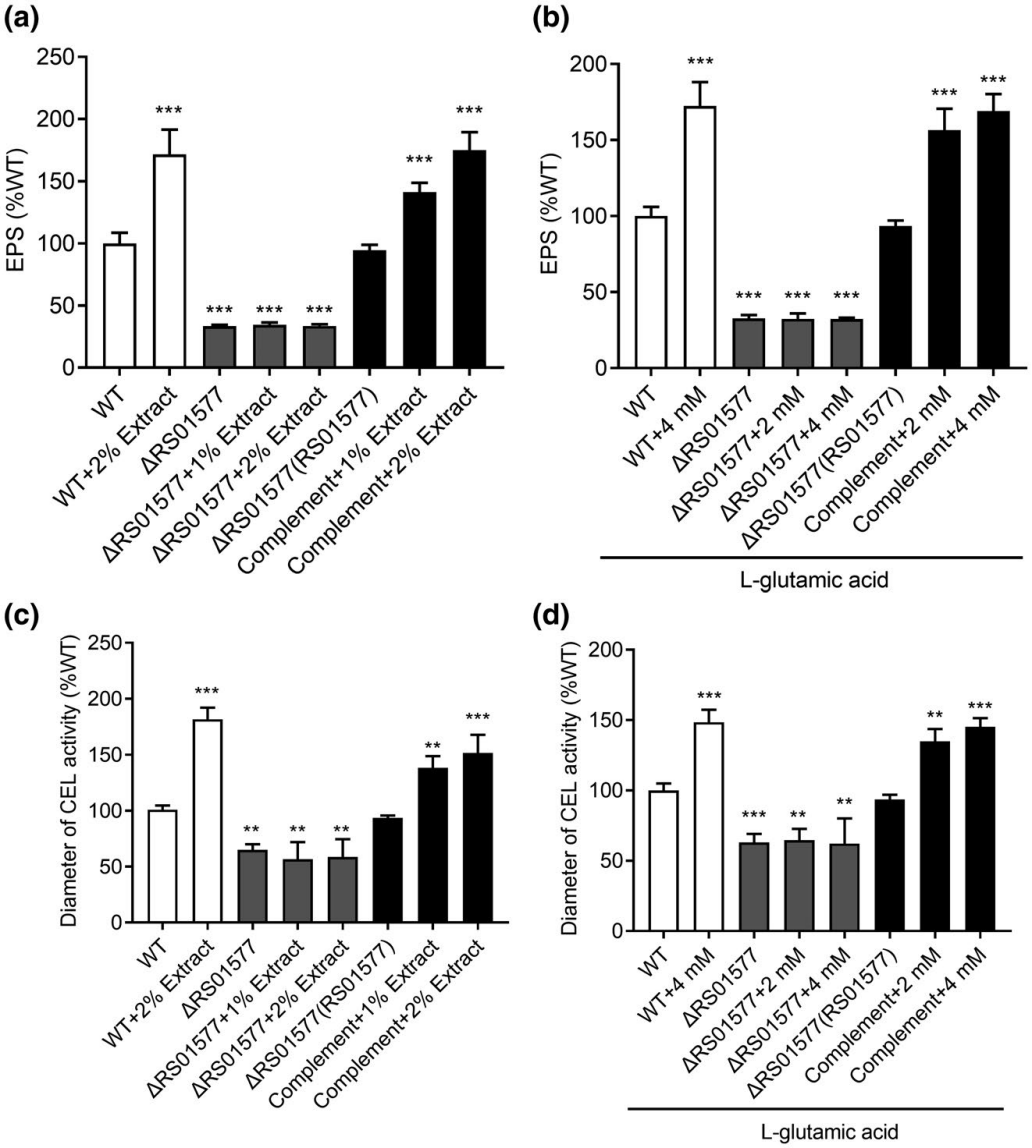
A hybrid sensor histidine kinase/response regulator is involved in the signalling of l‐glutamic acid. extracellular polysaccharide (EPS) production of the wild‐type (WT), the ΔRS01577 mutant, and the complementation strains supplemented with different concentrations of tomato extract (a) and l‐glutamic acid (b). Cellulase (CEL) activity of the wild‐type, the ΔRS01577 mutant, and the complementation strains supplemented with different concentrations of tomato extract (c) and l‐glutamic acid (d). The data are means ± *SD* of three independent experiments (unpaired *t* test, compared with the wild‐type strain GMI1000, **p < .01; ***p < .001)

To determine the regulatory components of the signalling pathway activated by plant‐derived l‐glutamic acid to regulate the expression of multiple virulence factors in *R. solanacearum*, we screened a Tn*5* insertion mutant library of *R. solanacearum* GMI1000 carrying an *epsA*‐*lacZ* promoter fusion plasmid for light blue colonies on Luria Bertani plates supplemented with X‐gal and l‐glutamic acid. We screened c.50,000 colonies and identified mutants with insertions in the known regulatory genes *phcA* and *pilT*. In addition, we identified a gene annotated as a hybrid sensor histidine kinase/response regulator (*RS01577*). RS01577 has four domains, namely, the HPT, H‐Kinase, CheA, and REC domains. An in‐frame deletion mutant of *RS01577* that we constructed exhibited reduced EPS production and cellulase activity, and the complemented strains restored the phenotypic changes (Figure 5). Notably, addition of tomato extract or l‐glutamic acid at different concentrations did not induce EPS production and cellulase activity in the *RS01577* deletion mutant (Figures 5 and S5), suggesting that RS01577 might be an essential component of the signalling pathway for the transduction of l‐glutamic acid to modulate virulence factor production in *R. solanacearum*.

### 2.6 RS01577 is a component of the signalling pathway of l‐glutamic acid

As deletion of *RS01577* completely abolished the induction of EPS production and cellulase activity by l‐glutamic acid in *R. solanacearum* GMI1000 (Figures 5 and S5), we continued to test the ability of l‐glutamic acid to enhance other biological functions, such as swimming motility and biofilm formation, in the *RS01577* deletion mutant. As described above, addition of 4 mM l‐glutamic acid considerably enhanced the motility and biofilm formation activity of the GMI1000 wild‐type strain (Figure 4e,f); however, addition of the same concentration of l‐glutamic acid to the *RS01577* deletion mutant also had a similar inducing effect as that observed for the wild‐type strain (Figure S6). These results indicate that there is more than one signalling pathway that can be activated by l‐glutamic acid, and RS01577 is one of the components of the signalling pathways of l‐glutamic acid to control biological functions and virulence factor production in *R. solanacearum* GMI1000.

### 2.7 Exogenous addition of l‐glutamic acid affects the expression levels of a wide range of genes in GMI1000

**Figure 6:**
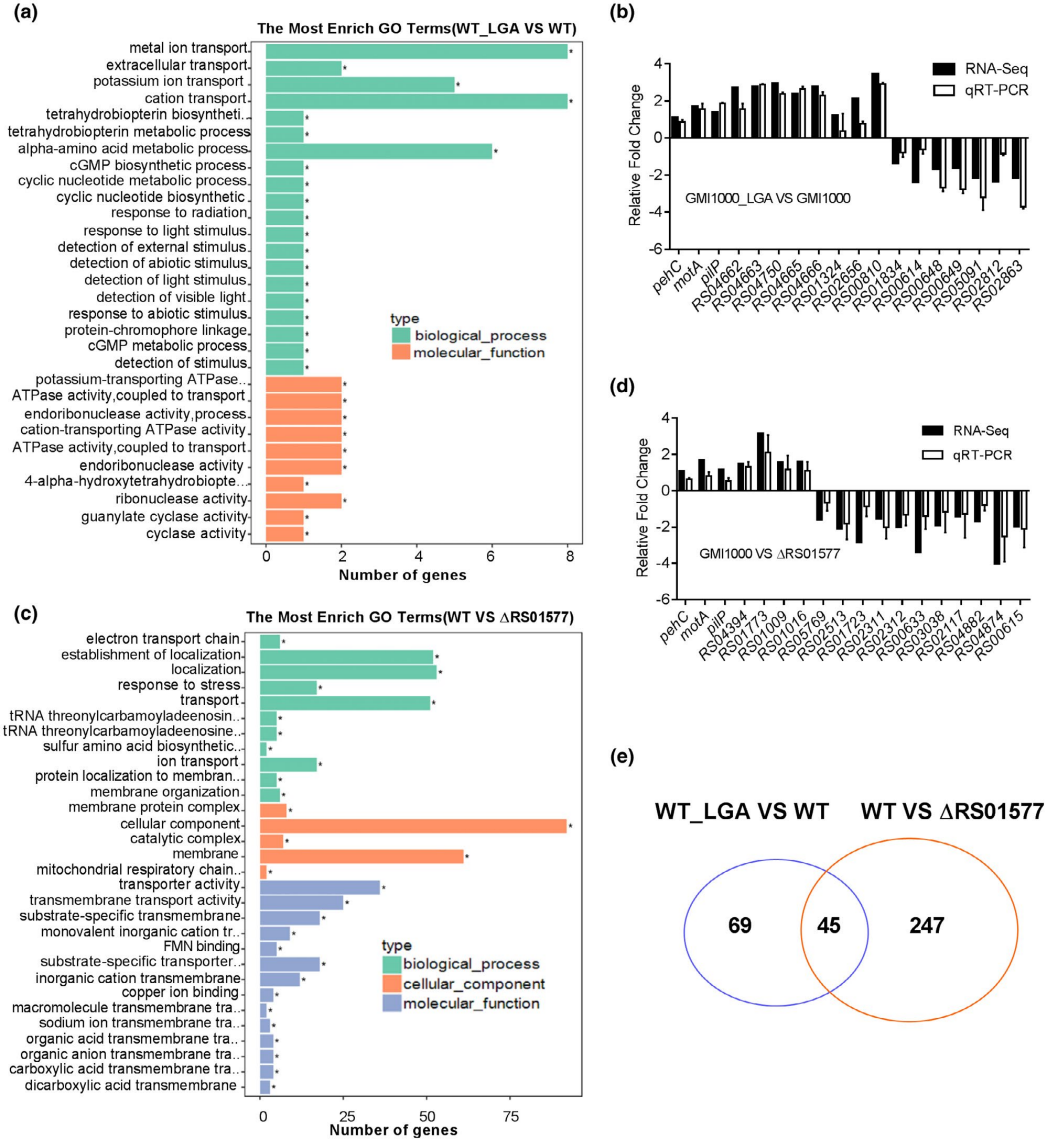
RS01577 and exogenous addition of l‐glutamic acid affect the expression levels of a wide range of genes in *Ralstonia solanacearum* GMI1000. Differential gene expression profiles between the wild‐type (WTR) strain GMI1000 in the absence and presence of l‐glutamic acid, and between the wild‐type GMI1000 and *RS01577* mutant, as measured by RNA‐Seq (log_2_ fold change ≥ 1). (a) Gene Ontology (GO) term enrichment analysis of differentially expressed genes between GMI1000_LGA and GMI1000. (b) Quantitative reverse transcription PCR (RT‐qPCR) analysis of the selected genes confirmed the RNA‐Seq results for GMI1000_LGA versus GMI1000. (c) GO term enrichment analysis of differentially expressed genes between GMI1000 and ΔRS01577. (d) RT‐qPCR analysis of the selected genes confirmed the RNA‐Seq results for GMI1000 versus ΔRS01577. (e) Venn diagrams showing the overlap of genes from different backgrounds. The abbreviation GMI1000_LGA indicates that the wild‐type GMI1000 strain was cultured in CPG medium supplemented with l‐glutamic acid

To determine the effect of l‐glutamic acid on the gene expression profile of *R. solanacearum*, we analysed and compared the transcriptomic profiles of the wild‐type GMI1000 strain cultured in the presence and absence of l‐glutamic acid using RNA‐Seq. Differential gene expression analysis showed that 114 genes exhibited altered expression (with a log_2_ fold change ≥ 1.0) in the wild‐type strain with the addition of 4 mM l‐glutamic acid (Figure 6A and Table S1). These differentially expressed genes are associated with a range of biological functions, including transcriptional regulation, membrane components, transport, signal transduction, motility, flagellum synthesis, stress tolerance, metabolism, and virulence (Figure 6a and Table S1). RT‐qPCR analysis of the selected genes confirmed the RNA‐Seq results (Figure 6b).

### 2.8 RS01577 affects the expression levels of a wide range of genes in GMI1000

To further investigate the roles of RS01577 in the regulation of bacterial physiology, we analysed and compared the transcriptomes of the wild‐type strain and the *RS01577* deletion mutant using RNA‐Seq. Differential gene expression analysis showed that 292 genes exhibited altered expression (with a log_2_ fold change ≥ 1.0) in the *RS01577* deletion mutant compared with the wild‐type strain, which we confirmed by RT‐qPCR analysis (Figure 6c,d and Table S1). These differentially expressed genes are also associated with various biological functions (Figure 6c and Table S1). We compared the profiles of the differentially expressed genes in the *RS01577* deletion mutant and the wild‐type strain after the addition of l‐glutamic acid and observed substantial overlap among these genes between the strains (Figure 6e and Table S1). Based on these observations, we concluded that RS01577 may play an important role in the intraspecies signalling of l‐glutamic acid in *R. solanacearum*.

### 2.9 Exogenous addition of tomato extract or l‐glutamic acid enhances *R. solanacearum* virulence toward tomato plants

**Figure 7:**
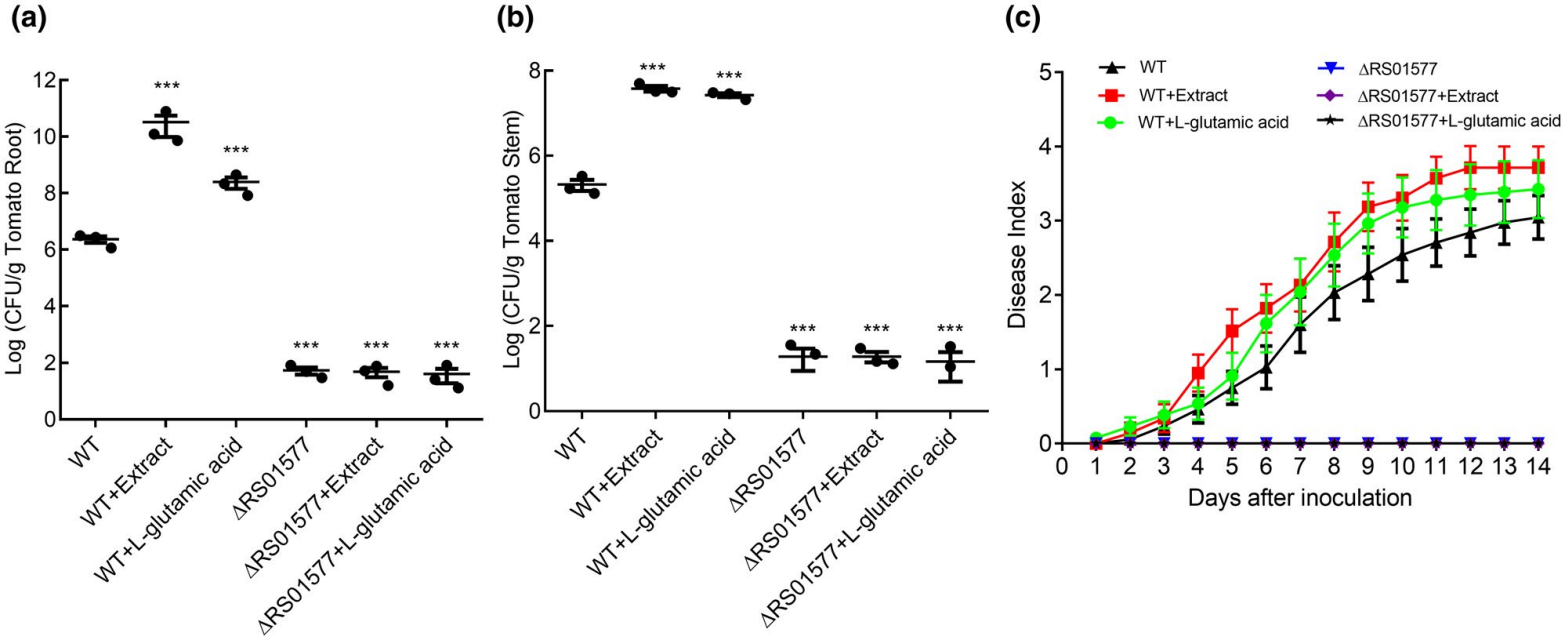
Exogenous addition of tomato extract or l‐glutamic acid enhances the virulence of *Ralstonia solanacearum* toward tomato plants. (a) Bacterial density in the root tissue was quantified by dilution analysis of root suspensions from inoculated plants. (b) *R. solanacearum* density in the stem was quantified by dilution of tissue suspensions, followed by plating 100 µl of the stem tissue suspension on CPG medium. (c) Effects of tomato extract and l‐glutamic acid on the disease index of bacterial wilt in tomato. Unwounded tomato plants were subjected to soil‐soak inoculation with 5 ml of bacterial culture (OD_600_ ≈ 1.0) and incubated at 28 ± 1 °C under a 14‐hr/10‐hr light/dark cycle. The data are means ± *SD* of three independent experiments (unpaired *t* test, compared with the wild‐type (WT) strain GMI1000, ***p < .001)

Given the strong induction of EPS production, cellulase activity, motility, and biofilm formation by tomato extract and l‐glutamic acid in *R. solanacearum*, the effect of the addition of tomato extract and l‐glutamic acid on the ability of *R. solanacearum* to infect host plants was also evaluated. We found that treatment with tomato extract or l‐glutamic acid significantly increased the wild‐type GMI1000 population in tomato roots at 5 days post‐inoculation (dpi; Figure 7a). Measurement of the GMI1000 colony‐forming units (cfu) in the inoculated plant roots revealed that addition of 2% tomato extract (per millilitre of culture) or 4 mM l‐glutamic acid increased the number of cfu from 2.7 × 10^6^ to 7.9 × 10^10^ or 2.5 × 10^8^, respectively, at 5 dpi (Figure 7a). Furthermore, exogenous addition of 2% tomato extract or 4 mM l‐glutamic acid also significantly promoted colonization to tomato stem by the wild‐type strain, and the number of cfu increased from 3.3 × 10^5^ to 3.8 × 10^7^ or 2.7 × 10^7^, respectively, at 5 dpi (Figure 7b). We continued to test the effect of tomato extract and l‐glutamic acid on the pathogenicity of *R. solanacearum* in tomato plants. Treatment with tomato extract or l‐glutamic acid considerably increased wilt symptoms in tomato plants and decreased the survival rate of the plants. In the absence of tomato extract and l‐glutamic acid, wilt symptoms appeared in tomato from 3 dpi with the wild‐type GMI1000. Treatment with tomato extract or l‐glutamic acid increased the disease index during the whole infection process (Figure 7c).

To further assess the role of *RS01577* in the pathogenicity of *R. solanacearum*, we also tested the cfu of the *RS01577* deletion mutant in the absence or presence of tomato extract and l‐glutamic acid in tomato roots and stems at 5 dpi. Deletion of *RS01577* resulted in a significant reduction in not only cell colonization but also pathogenicity in the host plants (Figure 7). Moreover, addition of neither tomato extract nor l‐glutamic acid could obviously enhance infection by the *RS01577* deletion mutant, suggesting an important role for RS01577 in both the pathogenesis of *R. solanacearum* and the transduction of l‐glutamic acid by the *R. solanacearum* wild‐type strain.

## 3 DISCUSSION

Host plant‐derived l‐glutamic acid can be used by *R. solanacearum* GMI1000, which represents a unique and fascinating mechanism by which the production and gene transcription of multiple virulence factors and the associated activities, such as EPS production, cellulase activity, biofilm formation, and motility, are triggered and boosted (Figures 4, 5 and 7). As expected, exogenous addition of tomato extract or l‐glutamic acid could also enhance the colonization ability of *R. solanacearum* in the roots and stems, and aggravate the disease index toward tomato (Figure 7). Previous studies have shown that glutamic acid, histidine, tryptophan, and aspartic acid are the main components of the xylem juice of tomato plants, and the concentration of l‐glutamic acid in plants is reduced significantly after infection of *R. solanacearum* (Wu *et al*., 2009; Zuluaga *et al*., 2013). Some findings have also suggested that cinnamic, myristic, and fumaric acids in tobacco roots induced plant infection by *R. solanacearum* (Li *et al*., 2017), suggesting that utilization of glutamic acid and other plant metabolic products is an important strategy adopted by *R. solanacearum* to promote infection.

Previous studies have indicated that exogenous glutamate can be used by rice via the SA signalling pathway to enhance inductive resistance to *Magnaporthe oryzae*, a semibiotrophic fungus that causes blast disease (Kadotani *et al*., 2016). Moreover, there is evidence that glutamate can be involved as a critical substrate associated with nitrogen and carbon metabolism in many bacteria and is supplied mainly by glutamate dehydrogenase (Wu *et al*., 2015). l‐glutamic acid was shown to be one key inductive compound that triggered the production of the main virulence factors and pathogenicity in *R. solanacearum* GMI1000 but had little effect on the growth rate of the pathogen cells (Figures 4 and S4), suggesting the existence of multiple and complex roles of l‐glutamic acid in the interaction between pathogens and plant hosts. Notably, the inductive effect of tomato extract was stronger than that of l‐glutamic acid, indicating that some other substances might work together with l‐glutamic acid.

Although the detailed mechanisms by which l‐glutamic acid aggravates bacterial wilt in tomato remain unclear, our results revealed that a two‐component hybrid sensor histidine kinase/response regulator is involved in signal perception or transduction to trigger the production of multiple virulence factors. Addition of l‐glutamic acid induced both virulence factor production and pathogenicity in the *R. solanacearum* GMI1000 wild‐type strain but had no detectable induction effect on EPS production, cellulase activity, or pathogenicity in the ΔRS01577 mutant (Figures 4, 5, 7, and S5). Moreover, the growth defect of ΔRS01577 in minimal medium might also contribute to the reduced pathogenicity of ΔRS01577 (Figure S7). Intriguingly, addition of tomato extract or l‐glutamic acid considerably enhanced the motility and biofilm formation of both the *R. solanacearum* GMI1000 wild‐type strain and the *RS01577* deletion mutant (Figure S6), demonstrating that there is more than one signalling pathway for the perception and transduction of l‐glutamic acid to induce biological functions in *R. solanacearum* GMI1000. Moreover, analysis of the transcriptomic profiles of the differentially expressed genes in the *RS01577* deletion mutant and the wild‐type strain after addition of l‐glutamic acid reflected only an overlap of target genes between the two transcriptomic profiles (Figure 6e and Table S1), demonstrating the complex pathways involved in the cross‐kingdom communication mediated by l‐glutamic acid.

## 4 EXPERIMENTAL PROCEDURES

### 4.1 Bacterial strains and culture conditions

The bacterial strains and plasmids used in this study are listed in Table 1. *Escherichia coli* strains were grown at 37 °C with shaking at 200 rpm in Lurai Bertani broth (1 L contained 10 g tryptone, 5 g yeast extract, and 10 g NaCl, pH 7.0). Tetracycline was used at 20 µg/ml for the *E. coli* strains. *R. solanacearum* GMI1000 (ATCCBAA‐1114) was obtained from the American Type Culture Collection (ATCC), and the Tn*5* mutant strains were grown at 28 °C on casamino acid‐peptone‐glucose (CPG) medium (Hendrick and Sequeira, 1984). Bacterial growth was determined by measuring optical density at a wavelength of 600 nm.

**Table 1:** Bacterial strains and plasmids used in this study

Strain/plasmid	Phenotypes and/or characteristics^a^	Reference or source
*Ralstonia solanacearum*		
GMI1000	Wild‐type strain of *R. solanacearum*	ATCCBAA‐1114
ΔRS01577	Deletion mutant with the *RS01577* gene being deleted	This study
ΔRS01577 (RS01577)	The complementation strain	This study
GMI1000 (P*epsA*‐*lacZ*)	GMI1000 harbouring the reporter construct P*epsA*‐*lacZ*	This study
ΔRS01577 (P*epsA*‐*lacZ*)	Mutant ΔRS01577 harbouring the reporter construct P*epsA*‐*lacZ*	This study
*Escherichia coli*		
DH5α	*supE44 lacU169(*ϕ80*lacZ*ΔM15*) hsdR17 recA1 endA1 gyrA96 thi‐1 relA1 pir*	Laboratory collection
**Plasmid**		
P*epsA*‐*lacZ*	pME2*‐lacZ* containing the *epsA* promoter, Tc^r^	This study
pBT20	Tn*5* transposon, Gm^r^	de Lorenzo *et al*. (1990)
pK18	pK18, *sacB*+; gene replacement vector; Kan^r^	Laboratory collection
pK18‐*RS01577*	pK18 containing fragments flanking *RS01577* and a Gm resistance fragment. Kan^r^, Gm^r^	This study

^a^ Tc^r^, Gm^r^, and Kan^r^ indicate resistance to tetracycline, gentamicin, and kanamycin, respectively.

The l‐glutamic acid (HPLC ≥ 99%) used in the study was purchased from Beijing TanMo Quality Testing Technology Co., Ltd. The compound was dissolved in hot distilled deionized (dd) H_2_O (100 °C) to a final concentration of 50 M and compound suspensions of different concentrations were prepared by adding hot ddH_2_O.

### 4.2 Construction of the Tn*5* transposon mutant library

A mini‐Tn*5* transposon derivative carrying a gentamicin resistance gene was electroporated into the wild‐type GMI1000 (De Lorenzo *et al*., 1990). Gentamicin‐resistant transformants were isolated after incubation on CPG medium containing 1% tomato extract and gentamicin, and were subsequently screened for mutant strains defective in EPS production. High‐efficiency thermal PCR was used to identify DNA flanking sequences at the insertion site of the Tn*5* transposon as previously described (Liu and Chen, 2007).

### 4.3 Construction of reporter strains and β‐galactosidase assay

The *epsA* promoter was amplified using the primer pair epsA‐F and epsA‐R, and then purified prior to ligation with the expression vector pME2‐*lacZ* digested with the same enzymes (Dong *et al*., 2008). The ligation vector verified by DNA sequencing was transformed into *R. solanacearum* by electroporation. The transformants were selected on CPG plates supplemented with gentamicin. The effect of tomato extract and l‐glutamic acid on *epsA* expression was determined by testing the β‐galactosidase activity of the pME2‐*lacZ* reporter gene as previously described (Zhang *et al*., 2011). The P*epsA*‐pME2‐*lacZ* reporter strain was inoculated in CPG medium for 12 hr with shaking at 200 rpm and 28 °C. The cells were inoculated into fresh CPG medium supplemented with different concentrations of tomato extract or l‐glutamic acid and cultured for 6–7 hr with shaking at 200 rpm and 28 °C. When the OD_600_ reached 1.0, the β‐galactosidase activity was measured as previously described (Zhou *et al*., 2007).

### 4.4 Construction of the in‐frame deletion mutant

*R. solanacearum* GMI1000 was used as the parental strain for the generation of in‐frame deletion mutants by following previously described methods (Boon *et al*., 2008). The primers used to generate the upstream and downstream flanking regions are listed in Table S2.

### 4.5 Analysis of cellulase activity

Cellulase activity was tested in cellulase detection medium (1 g carboxymethylcellulose, 3.8 g Na_3_PO_4_, 8 g agarose, pH 7.0, per litre). Different concentrations of tomato extract or l‐glutamic acid were added to the medium. Overnight culture was diluted to an OD_600_ of approximately 0.01 and 2‐μl bacterial suspensions were plotted on the growth plate containing tomato extract or l‐glutamic acid for measurement of cellulase activity. The plates were incubated at 28 °C for 48 hr. Colonies grown on cellulase detection medium were stained with 0.5% Congo red for 30 min, and the plates were incubated with 1 M NaCl solution for 10–15 min at room temperature (Barras *et al*., 1987; Chatterjee *et al*., 1995). After discarding the solution, cellulase activity in the plates was measured. Each treatment was replicated at least three times.

### 4.6 EPS quantification assay

Measurement of EPS production was performed as previously reported with some modifications (Zhu *et al*., 2010). Briefly, bacteria were cultured in sucrose, peptone, and agar (SPA) liquid medium (1 L contains 5 g peptone, 20 g sucrose, 0.5 g KH_2_PO_4_, and 0.25 g MgSO_4_, pH 7.2) supplemented with different concentrations of tomato extract (from 0.5% to 2%) or l‐glutamic acid (from 1 to 4 mM) to an OD_600_ of 3.0. Then a 100‐ml aliquot of the culture was collected and centrifuged at 12,000 rpm for 20 min and the supernatants were filtered through a 0.22 μm membrane. After mixing the collected supernatants with 4 volumes of absolute ethanol, the mixture was incubated at 4 °C overnight. The precipitated EPS was isolated by centrifugation and dried overnight at 55 °C before the determination of dry weight.

### 4.7 Swimming motility assay

The swimming motility of *R. solanacearum* was determined on semisolid medium containing 0.8% tryptone and 0.35% agar (Becton, Dickinson and Co.) (Kelman and Hruschka, 1973). Different concentrations of tomato extract or l‐glutamic acid were added to the semisolid motility medium, the overnight culture was diluted to an OD_600_ of 0.01, and 2‐μl bacterial suspensions were dropped into the centres of the growth plates. The halo diameters were measured after culturing for 48 hr at 28 °C. The experiment was repeated three times.

### 4.8 Biofilm formation assay

Measurement of biofilm formation by *R. solanacearum* was performed in 96‐well polystyrene plates as previously reported, with some modifications (Yao and Allen, 2007). Briefly, an overnight bacterial culture was resuspended in CPG medium supplemented with different concentrations of tomato extract (from 0.5% to 2%) or l‐glutamic acid (from 1 to 4 mM) and was precisely adjusted to an OD_600_ of 0.05. The samples were statically incubated at 28 °C for 16 hr. After staining with 0.01% crystal violet and dissolving the stain in 95% ethanol, biofilm formation was quantified by measuring the absorbance at 570 nm (A_570_).

### 4.9 Purification and structural analysis of the active compound

To isolate and identify the active component from tomato extract, 2.5 kg tomato fruit was squeezed and extracted with 1 L ethanol for 24 hr at 4 °C twice. The sample was centrifuged and the supernatant was concentrated by a rotary evaporator with heat to 40 °C under vacuum. The residue was then dissolved with methanol:water (1:1) to a volume of 250 ml and eluted by HPLC on a C18 reverse‐phase column (XBridge, 10 µm, 19 × 250 mm) with an acetonitrile:water gradient from 10:90 to 100:0 (vol/vol) at a flow rate of 7 ml/min for 30 min. The elute was fractionated and tested for induction activity on EPS production and cellulase activity. The active fraction from the tomato extract was detected and purified by HPLC using a semipreparative C18 reverse‐phase column again (XBridge, 10 µm, 19 × 250 mm) with an acetonitrile:water gradient from 20:80 to 50:50 (vol/vol) at a flow rate of 3 ml/min. Fractions were collected to test for induction activity on EPS production and cellulase activity. The active fraction from the tomato extract was further detected and purified by HPLC using a semipreparative C18 reverse‐phase column (XBridge, 10 µm, 19 × 250 mm) with acetonitrile:water at 30:70 (vol/vol) at a flow rate of 3 ml/min. The active fraction was purified by semipreparative HPLC (XBridge, 10 µm, 19 × 250 mm) using acetonitrile:water (30:70, vol/vol).

To identify the structure of the compound, a Bruker AV‐500 (Bruker Instrument, Inc.) spectrometer was used to obtain the ^1^H (500 MHz) and ^13^C (125 MHz) NMR spectra in CD_3_OD solution (Song *et al*., 2018).

### 4.10 Bacterial growth analysis

An overnight bacterial culture in CPG medium was inoculated into fresh CPG medium or minimal medium (K_3_PO_4_, 60 mM; KH_2_PO_4_, 30 mM; citrate, 20 mM; (NH_4_)_2_SO_4_, 15 mM; MgSO_4_.7H_2_O, 0.8 mM; CaCl_2_, 90 mM; FeSO_4_, 30 mM; MnCl_2_, 15 mM; casamino acids, 0.5%) (Boon *et al*., 2008) to an OD_600_ of 0.01, and then different amounts of tomato extract and l‐glutamic acid were added. A 200‐μl cell suspension was grown in each well at 28 °C in a low‐intensity shaking model using Bioscreen‐C automated growth curve analysis system. CPG medium and minimal medium were used as the negative control.

### 4.11 Virulence assay

Wilt‐susceptible tomato plants (cultivar Xinjinfeng No. 1) were used for the virulence test. The root‐drenching method as described by Kanda *et al*. (2003) was used to evaluate the virulence of *R. solanacearum*. We used CPG broth without bacteria as the negative control, and different concentrations of tomato extract or l‐glutamic acid were added directly to the bacterial cultures. Each plant was inoculated by pouring 5 ml of a bacterial suspension with an OD_600_ of approximately 1.0 into soil close to the plant roots. Inoculated plants were placed in a cabinet with a 14‐hr/10‐hr light/dark cycle at 28 °C. The inoculation experiments were repeated three times, and 14 plants were used for each group each time. All the plants were monitored for disease index analysis, and the following scale was used: 0, no symptoms; 1, 1%–25% of leaves wilted; 2, 26%–50% of leaves wilted; 3, 51%–75% of leaves wilted; 4, 76%–100% of leaves wilted (Yang *et al*., 2017). At 5 dpi, 1 g of tissues from the plant roots and stems were collected, milled in a sterile mortar with 9 ml of sterile water and diluted in gradient. The diluted suspensions were plated on CPG plates to determine the bacterial cfu in the tomato roots and stems.

### 4.12 RNA‐Seq and RT‐qPCR analysis

Total RNA was isolated from *R. solanacearum* GMI1000 (OD_600_ ≈ 1.0) strains using the Eastep Super Total RNA Extraction Kit (Promega). cDNA synthesis and high‐throughput RNA‐Seq were performed as described previously (An *et al*., 2014). Trimmed sequence reads were aligned to the *R. solanacearum* GMI1000 genome sequence using Bowtie2 v. 2.2.3 (Langmead and Salzberg, 2012). HTSeq v. 0.6.1 was used to count the read numbers mapped to each gene, and the fragments per kilobase of transcript per million reads mapped (FPKM) of each gene was calculated based on the length of the gene and read counts mapped to the gene (Trapnell *et al*., 2010).

cDNA synthesis and RT‐qPCR analysis were performed with the ChamQ Universal SYBR qPCR Master Mix (Vazyme) on a 7300 Plus real‐time PCR system (Applied Biosystems) following the manufacturer's instructions. The gene expression level of *recA* was used as a control for RT‐qPCR analysis. The 2^−ΔΔCt^ method was used to calculate the relative expression of target genes (Livak and Schmittgen, 2001; Jacobs *et al*., 2012). The primers used in RT‐qPCR analysis are listed in Table S2.

### 4.13 Analysis of amino acid concentration

Quantitative analysis of l‐glutamic acid in the tomato extract was carried out using an automated amino acid analyzer (S433D; Sykam) following the method previously described in GB/T 30987‐2014. l‐glutamic acid was analysed on an ion exchange column (LCA K07/Li, Peek, 4.6 × 100 mm) and the detection wavelength was reduced to 440 nm from 570 nm. The mobile phase was lithium citrate (A: pH 2.9; B: pH 4.2; C: pH 8.0) and regeneration solution D (isopropanol:ultrapure water at 30:70 [vol/vol]) at a flow rate of 0.45 ml/min. The ninhydrin solution was added to the water at a flow rate of 0.25 ml/min. The column temperature gradient was from 41 to 74 °C (Hohmann *et al*., 2014). The concentration of l‐glutamic acid in tomato extract was determined by analysis of the peak area compared with that of standard samples.

### 4.14 Statistical analysis

Statistical analysis was performed with Prism 8 software (GraphPad). The data are presented as the means ± standard deviations of three independent experiments. Student's *t* test was performed with statistical significance set at the 0.05 confidence level.

## Supporting information


**FIGURE S1** Growth curves of *Ralstonia solanacearum* wild‐type GMI1000 strain supplemented with different concentrations of tomato extract. The data shown are the means of three independent experiments, and error bars indicate the *SD*

**FIGURE S2** Analysis of the active fractions of tomato extract. (a) Separation of active compounds from tomato extract by HPLC. (b) Effects of different fractions on the cellulase activity of *Ralstonia solanacearum* GMI1000

**FIGURE S3** Quantitative analysis of l‐glutamic acid in tomato extract using amino acid automatic analyzer. (a) The peak of the mixed standard samples. (b) The peak of the diluted tomato extract sample. Red arrows point to the peaks of l‐glutamic acid

**FIGURE S4** Growth curves of *Ralstonia solanacearum* wild‐type GMI1000 strain with addition of different concentrations of l‐glutamic acid. The data shown are the means of three independent experiments and error bars indicate the *SD*

**FIGURE S5** Effects of tomato extract and l‐glutamic acid on cellulase activity of *Ralstonia solanacearum* wild‐type strain and *RS01577* mutant strain. (a) The cellulase activity of *R. solanacearum* wild‐type strain with addition of different amounts of tomato extract. (b) The cellulase activity of *R. solanacearum* wild‐type strain supplemented with different concentrations of l‐glutamic acid. (c) The cellulase activity of *R. solanacearum* *RS01577* mutant strain with addition of different amounts of tomato extract. (d) The cellulase activity of *R. solanacearum* *RS01577* mutant strain supplemented with different concentrations of l‐glutamic acid.

**FIGURE S6** Effects of tomato extract and l‐glutamic acid on the swimming motility (a), (b) and biofilm formation (c), (d) of *Ralstonia solanacearum* GMI1000, respectively. The data shown are the means of three independent experiments and error bars indicate the *SD*

**FIGURE S7** Growth curves of wild‐type *Ralstonia solanacearum* GMI1000, the *RS01577* mutant strain, and the complemented strain in CPG medium (a) and minimal medium (b). The data shown are the means of three independent experiments and error bars indicate the *SD*

**TABLE S1** List of genes differentially expressed in the wild‐type *Ralstonia solanacearum* GMI1000 and the Δ*RS01577* mutant strain, and the wild‐type GMI1000 strain in the presence and absence of l‐glutamic acid (log_2_ fold change ≥1.0)

**TABLE S2** PCR primers used in this study

## Data Availability

The data that support the findings of this study are available from the corresponding author upon reasonable request.
