# Supplementary material for: Ralstonia solanacearum promotes pathogenicity by utilizing l‐glutamic acid from host plants
Source: Mol Plant Pathol. 2020 Jun 29;21(8):1099–110. doi: 10.1111/mpp.12963 (PMC7368120; doi:10.1111/mpp.12963)
Supplement: Supplementary file 2 — FIGURE S2 Analysis of the active fractions of tomato extract. (a) Separation of active compounds from tomato extract by HPLC. (b) Effects of different fractions on the cellulase activity of Ralstonia solanacearum GMI1000 [file MPP-21-1099-s002.docx]

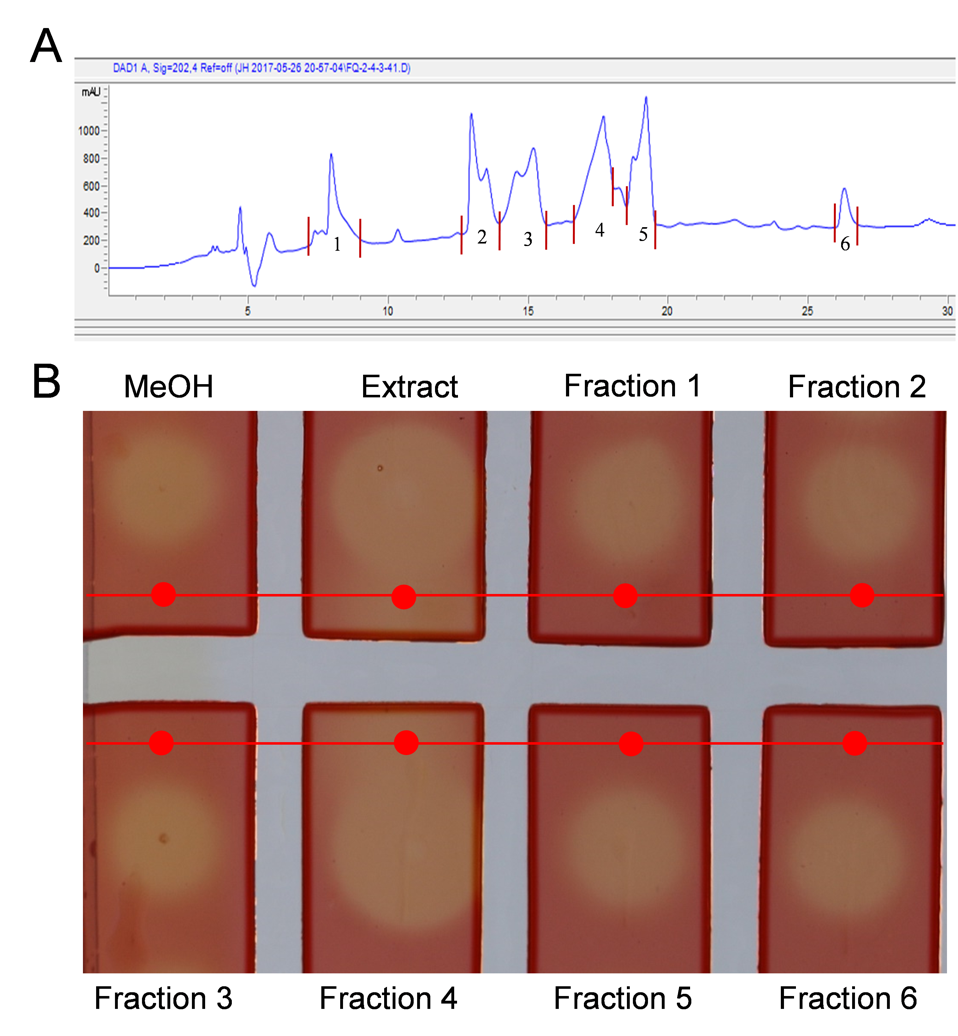


**Fig S2.** Analysis of the active fractions of tomato extract. (A) Separation of active compounds from tomato extract by HPLC. (B) Effects of different fractions on the cellulase activity of *R. solanacearum* GMI1000.
