# Supplementary material for: Ralstonia solanacearum promotes pathogenicity by utilizing l‐glutamic acid from host plants
Source: Mol Plant Pathol. 2020 Jun 29;21(8):1099–110. doi: 10.1111/mpp.12963 (PMC7368120; doi:10.1111/mpp.12963)
Supplement: Supplementary file 3 — FIGURE S3 Quantitative analysis of l‐glutamic acid in tomato extract using amino acid automatic analyzer. (a) The peak of the mixed standard samples. (b) The peak of the diluted tomato extract sample. Red arrows point to the peaks of l‐glutamic acid [file MPP-21-1099-s003.docx]

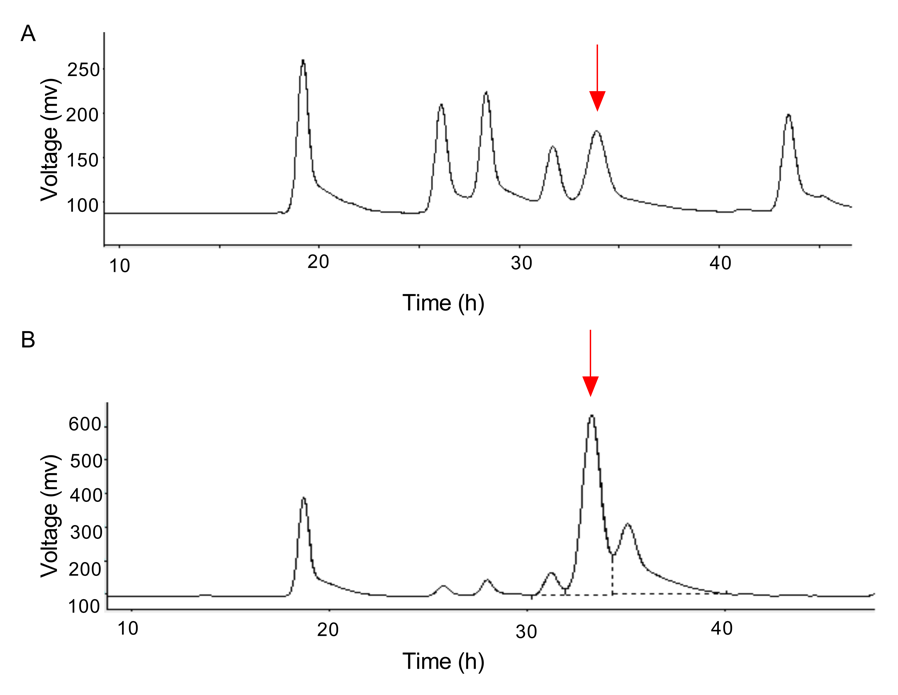


**Fig S3.** Quantitative analysis of L-glutamic acid in tomato extract by using amino acid automatic analyzer. (A) The peak of the mixed standard samples. (B) The peak of the diluted tomato extract sample. Red arrows point to the peaks of L-glutamic acid.
