# Supplementary material for: Ralstonia solanacearum promotes pathogenicity by utilizing l‐glutamic acid from host plants
Source: Mol Plant Pathol. 2020 Jun 29;21(8):1099–110. doi: 10.1111/mpp.12963 (PMC7368120; doi:10.1111/mpp.12963)
Supplement: Supplementary file 4 — FIGURE S4 Growth curves of Ralstonia solanacearum wild‐type GMI1000 strain with addition of different concentrations of l‐glutamic acid. The data shown are the means of three independent experiments and error bars indicate the SD [file MPP-21-1099-s004.docx]

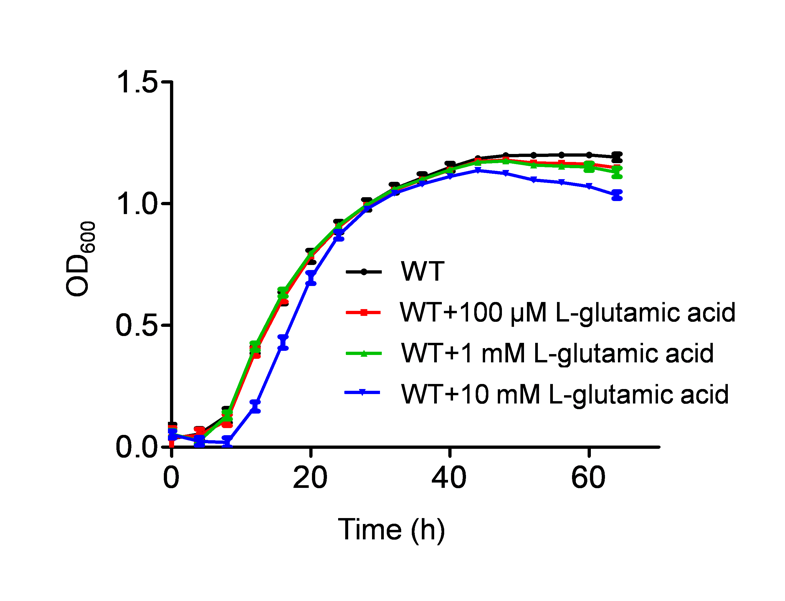


**Fig S4.** Growth curves of *R. solanacearum* wild-type GMI1000 strain with addition of different concentrations of L-glutamic acid. The data shown are the means of three independent experiments, and error bars indicate the SDs.
