# Supplementary material for: Ralstonia solanacearum promotes pathogenicity by utilizing l‐glutamic acid from host plants
Source: Mol Plant Pathol. 2020 Jun 29;21(8):1099–110. doi: 10.1111/mpp.12963 (PMC7368120; doi:10.1111/mpp.12963)
Supplement: Supplementary file 5 — FIGURE S5 Effects of tomato extract and l‐glutamic acid on cellulase activity of Ralstonia solanacearum wild‐type strain and RS01577 mutant strain. (a) The cellulase activity of R. solanacearum wild‐type strain with addition of different amounts of tomato extract. (b) The cellulase activity of R. solanacearum wild‐type strain supplemented with different concentrations of l‐glutamic acid. (c) The cellulase activity of R. solanacearum RS01577 mutant strain with addition of different amounts of tomato extract. (d) The cellulase activity of R. solanacearum RS01577 mutant strain supplemented with different concentrations of l‐glutamic acid. [file MPP-21-1099-s005.docx]

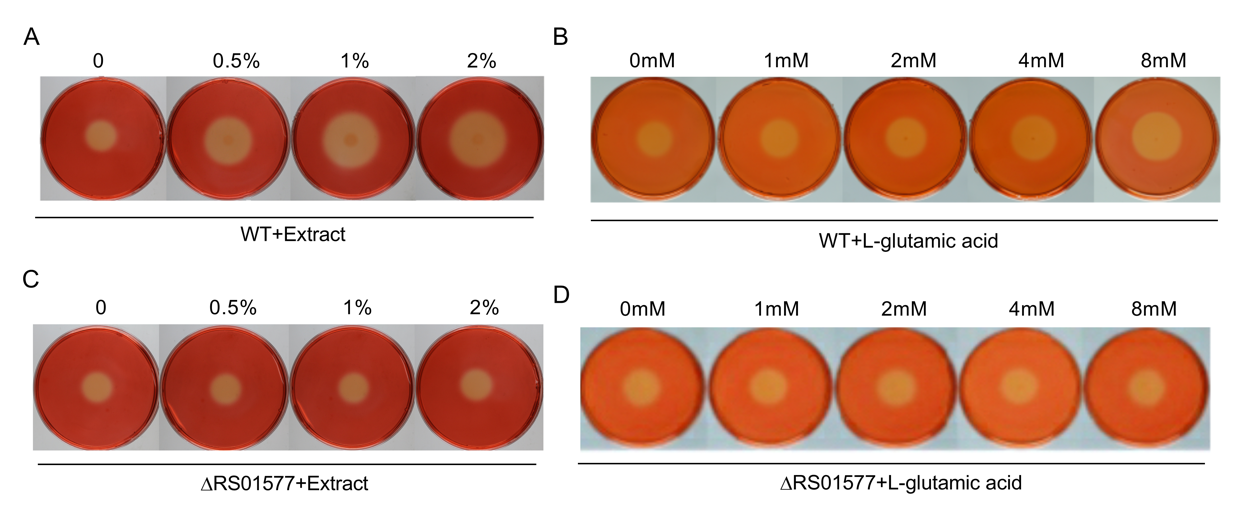


**Fig S5.** Effects of tomato extract and L-glutamic acid on cellulase activity of *R. solanacearum* wild-type strain and *RS01577* mutant strain. (A) The cellulase activity of *R. solanacearum* wild-type strain with addition of different amounts of tomato extract. (B) The cellulase activity of *R. solanacearum* wild-type strain supplemented with different concentrations of L-glutamic acid. (C) The cellulase activity of *R. solanacearum* *RS01577* mutant strain with addition of different amounts of tomato extract. (D) The cellulase activity of *R. solanacearum* *RS01577* mutant strain supplemented with different concentrations of L-glutamic acid.
