# Supplementary material for: Ralstonia solanacearum promotes pathogenicity by utilizing l‐glutamic acid from host plants
Source: Mol Plant Pathol. 2020 Jun 29;21(8):1099–110. doi: 10.1111/mpp.12963 (PMC7368120; doi:10.1111/mpp.12963)
Supplement: Supplementary file 6 — FIGURE S6 Effects of tomato extract and l‐glutamic acid on the swimming motility (a), (b) and biofilm formation (c), (d) of Ralstonia solanacearum GMI1000, respectively. The data shown are the means of three independent experiments and error bars indicate the SD [file MPP-21-1099-s006.docx]

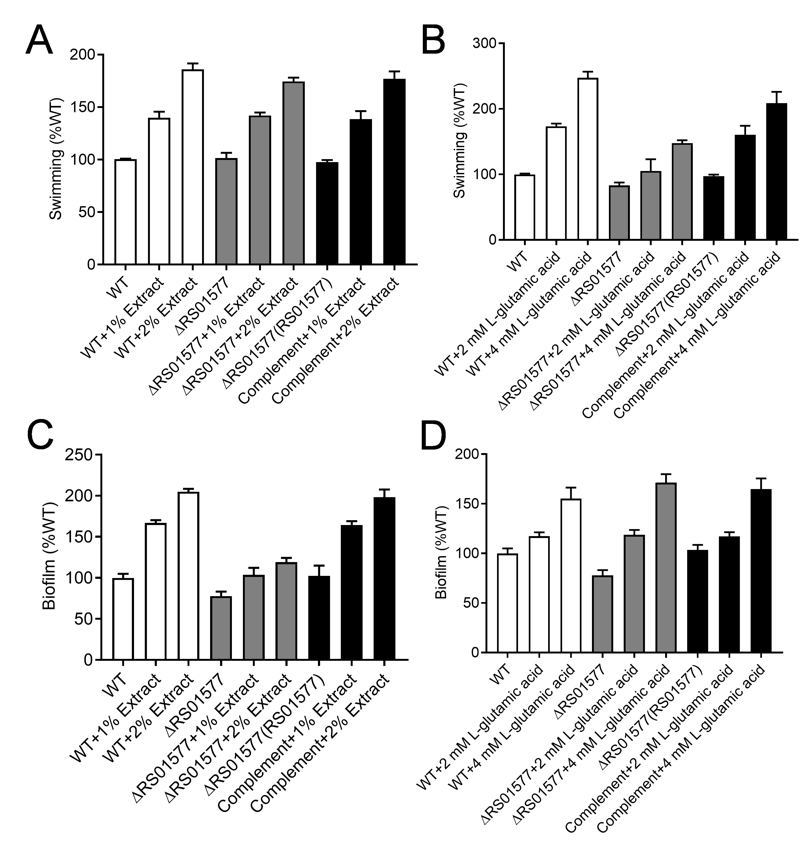


**Fig S6.** Effects of tomato extract and L-glutamic acid on the swimming motility (A, B) and biofilm formation (C, D) of *R. solanacearum* GMI1000 strains, respectively. The data shown are the means of three independent experiments, and error bars indicate the SDs.
