# Supplementary material for: Ralstonia solanacearum promotes pathogenicity by utilizing l‐glutamic acid from host plants
Source: Mol Plant Pathol. 2020 Jun 29;21(8):1099–110. doi: 10.1111/mpp.12963 (PMC7368120; doi:10.1111/mpp.12963)
Supplement: Supplementary file 7 — FIGURE S7 Growth curves of wild‐type Ralstonia solanacearum GMI1000, the RS01577 mutant strain, and the complemented strain in CPG medium (a) and minimal medium (b). The data shown are the means of three independent experiments and error bars indicate the SD [file MPP-21-1099-s007.docx]

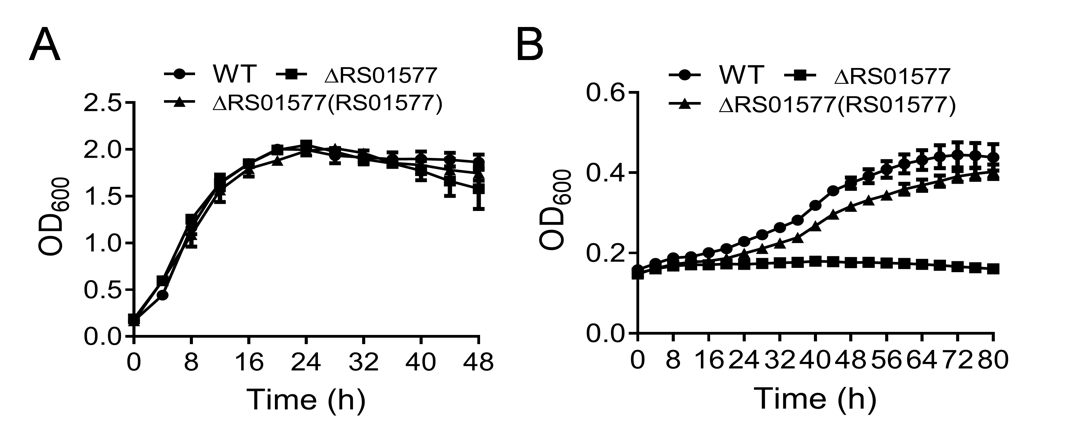


**Fig S7.** Growth curves of the *R. solanacearum* wild-type GMI1000 strain, the *RS01577* mutant strain and the complement strain in CPG medium (A) and minimal medium (B). The data shown are the means of three independent experiments, and error bars indicate the SDs.
