# Supplementary material for: Ralstonia solanacearum promotes pathogenicity by utilizing l‐glutamic acid from host plants
Source: Mol Plant Pathol. 2020 Jun 29;21(8):1099–110. doi: 10.1111/mpp.12963 (PMC7368120; doi:10.1111/mpp.12963)
Supplement: Supplementary file 8 — TABLE S1 List of genes differentially expressed in the wild‐type Ralstonia solanacearum GMI1000 and the ΔRS01577 mutant strain, and the wild‐type GMI1000 strain in the presence and absence of l‐glutamic acid (log2 fold change ≥1.0) [file MPP-21-1099-s008.doc]

**Table S1. List of genes differentially expressed in the wild-type GMI1000 strain and** **the ΔRS01577 mutant strain, and the wild-type GMI1000 strain in the presence and absence of L-glutamic acid (Log_2_ fold change ≥1.0).**

| **Gene ID** | **Gene name^a^** | **Description** | **Log_2_ fold change** | |
| --- | --- | --- | --- | --- |
|  |  |  | **GMI1000_LGA vs GMI1000** | **GMI1000 vs ΔRS01577** |
| RS_RS22045 | *epsA* | EPS I polysaccharide export outer membrane protein EpsA | 1.309 | 0.639 |
| RS_RS22020 | *epsE* | EPS I polysaccharide export inner membrane protein EpsE | 2.203 | -0.692 |
| RS_RS22015 | *epsF* | EPS I polysaccharide export inner membrane protein EpsF | 1.457 | 0.457 |
| RS_RS23935 | *motA* | flagellar motor stator protein MotA | 1.720 | 1.72 |
| RS_RS14895 | *pilP* | pilus assembly protein PilP | 1.368 | 1.17 |
| RS_RS14900 | *pilO* | type IV pilus biogenesis protein PilO | 1.2121 |  |
| RS_RS18995 |  | flagellin | 3.4491 |  |
| RS_RS21105 | *pehC* | endopolygalacturonase | 1.101 | 1.101 |
| RS_RS17910 | - | PhoPQ-regulated protein | 1.2722 | - |
| RS_RS00055 | - | TetR family transcriptional regulator | -1.3599 | - |
| RS_RS00110 | - | carbon starvation protein A | 1.893 | - |
| RS_RS00115 | - | DUF466 domain-containing protein | 1.4586 | - |
| RS_RS00535 | - | thiazole synthase | 1.0583 | - |
| RS_RS00560 | - | nitronate monooxygenase | -2.2739 | - |
| RS_RS00580 | - | hypothetical protein | 1.0407 | - |
| RS_RS00780 | - | arginase | -1.2864 | - |
| RS_RS00785 | *rocD* | ornithine--oxo-acid transaminase | -1.2808 | - |
| RS_RS00895 | - | DUF4010 domain-containing protein | -1.9067 | -2.3419 |
| RS_RS00905 | - | CBS domain-containing protein | -2.3551 | -2.5992 |
| RS_RS00955 | - | hypothetical protein | -2.1878 | -2.4869 |
| RS_RS01070 | - | MFS transporter | 1.666 | 2.1328 |
| RS_RS01075 | - | aldo/keto reductase | -1.6045 | 2.7681 |
| RS_RS01140 | - | hypothetical protein | -1.8224 | -1.5066 |
| RS_RS01595 | - | DUF2917 domain-containing protein | -1.346 | - |
| RS_RS02740 | - | MFS transporter | 1.4695 | - |
| RS_RS03840 | - | DUF3079 domain-containing protein | -2.151 | -1.577 |
| RS_RS03905 | - | hemerythrin | -1.6652 | -1.0652 |
| RS_RS06020 | - | alkene reductase | 1.1259 | - |
| RS_RS06385 | - | copper-translocating P-type ATPase | -2.3372 | - |
| RS_RS06395 | *ccoN* | cytochrome-c oxidase%2C cbb3-type subunit I | -1.8922 | - |
| RS_RS06415 | *ccoG* | cytochrome c oxidase accessory protein CcoG | 1.3383 | -1.309 |
| RS_RS06425 | - | hypothetical protein | 3.8267 | - |
| RS_RS06435 | - | membrane protein | 2.1425 | - |
| RS_RS06725 | - | aliphatic sulfonate ABC transporter ATP-binding protein | 2.1374 | - |
| RS_RS06750 | *cysW* | sulfate ABC transporter permease subunit CysW | 1.9115 | -1.1442 |
| RS_RS06755 | - | sulfate/thiosulfate import ATP-binding protein CysA | 1.5793 | -1.0503 |
| RS_RS06905 | - | MurR/RpiR family transcriptional regulator | 2.6235 | - |
| RS_RS06910 | - | isoaspartyl peptidase/L-asparaginase | 2.7918 | - |
| RS_RS06915 | - | ABC transporter ATP-binding protein | 2.7189 | - |
| RS_RS06920 | - | glutathione ABC transporter substrate-binding protein GsiB | 2.7713 | - |
| RS_RS06925 | - | glutathione ABC transporter permease GsiC | 2.9267 | - |
| RS_RS06930 | - | glutathione ABC transporter permease GsiD | 2.3941 | - |
| RS_RS06935 | - | S58 family peptidase | 2.7696 | - |
| RS_RS06940 | - | D-aminopeptidase | 2.946 | - |
| RS_RS07560 | - | universal stress protein | 2.8893 | -1.9203 |
| RS_RS07580 | - | hypothetical protein | -1.2055 | -1.6905 |
| RS_RS07590 | - | DUF1488 domain-containing protein | -1.5246 | -1.8415 |
| RS_RS08055 | *aceE* | pyruvate dehydrogenase (acetyl-transferring)%2C homodimeric type | 1.1111 | - |
| RS_RS09165 | - | siderophore biosynthesis protein | 1.228 | -1.1649 |
| RS_RS10035 | - | GntR family transcriptional regulator | -1.3564 | - |
| RS_RS11100 | - | MBL fold metallo-hydrolase | -2.3609 | -1.0916 |
| RS_RS11355 | - | D-amino acid dehydrogenase small subunit | 1.8572 | - |
| RS_RS13240 | - | imidazolonepropionase | -1.9219 | - |
| RS_RS13245 | - | formimidoylglutamase | -2.1248 | - |
| RS_RS13250 | *hutH* | histidine ammonia-lyase | -1.549 | - |
| RS_RS13255 | - | urocanate hydratase | -1.1307 | 1.2143 |
| RS_RS14855 | *gltD* | glutamate synthase subunit beta | 1.3804 | - |
| RS_RS14860 | - | glutamate synthase subunit alpha | 1.2601 | - |
| RS_RS14870 | - | OmpW family protein | -2.5399 | -1.5531 |
| RS_RS14905 | - | ferrous iron transporter B | 1.157 | - |
| RS_RS15525 | - | porin | 1.0778 | - |
| RS_RS15585 | *hppD* | 4-hydroxyphenylpyruvate dioxygenase | -1.0865 | - |
| RS_RS15680 | - | MFS transporter | 1.1001 | 1.4821 |
| RS_RS16455 | *hemN* | oxygen-independent coproporphyrinogen III oxidase | -2.8357 | -2.0984 |
| RS_RS16465 | - | acyl-homoserine-lactone synthase | 1.3182 | - |
| RS_RS16475 | - | sugar-binding lectin protein | -1.8148 | - |
| RS_RS16500 | *gcvT* | aminomethyltransferase | -1.0951 | - |
| RS_RS16510 | *gcvP* | glycine dehydrogenase | -1.1142 | - |
| RS_RS16515 | - | L-serine ammonia-lyase | -1.144 | - |
| RS_RS16810 | - | phenylalanine 4-monooxygenase | -1.7839 | - |
| RS_RS16815 | - | 4a-hydroxytetrahydrobiopterin dehydratase | -1.3902 | - |
| RS_RS16930 | - | hypothetical protein | 1.4788 | - |
| RS_RS16940 | *kdpF* | K(+)-transporting ATPase subunit F | 1.9097 | - |
| RS_RS16945 | - | potassium-transporting ATPase A chain | 2.1095 | - |
| RS_RS16950 | *kdpB* | potassium-transporting ATPase subunit B | 2.6825 | - |
| RS_RS16955 | - | potassium-transporting ATPase subunit C | 2.5804 | - |
| RS_RS16960 | - | sensor histidine kinase KdpD | 2.0031 | - |
| RS_RS17635 | - | hypothetical protein | 3.3225 | - |
| RS_RS17725 | *prpF* | 2-methylaconitate cis-trans isomerase PrpF | -5.379 | 4.2873 |
| RS_RS17730 | *acnD* | Fe/S-dependent 2-methylisocitrate dehydratase AcnD | -4.5157 | 4.3617 |
| RS_RS17735 | - | 2-methylcitrate synthase | -4.8321 | 4.5466 |
| RS_RS17740 | - | methylisocitrate lyase | -4.6583 | 5.297 |
| RS_RS18680 | - | heavy metal translocating P-type ATPase | -1.2903 | - |
| RS_RS19095 | - | hypothetical protein | -2.1363 | -1.4972 |
| RS_RS19480 | - | ornithine cyclodeaminase | -1.2241 | - |
| RS_RS19485 | - | hypothetical protein | -1.517 | - |
| RS_RS19515 | - | hypothetical protein | -1.1909 | 1.3726 |
| RS_RS19545 | - | DUF2087 domain-containing protein | -1.2502 | - |
| RS_RS19555 | - | class I SAM-dependent methyltransferase | -1.3126 | - |
| RS_RS19560 | - | MFS transporter | 1.2663 | 1.175 |
| RS_RS19565 | - | hypothetical protein | -1.1327 | 1.1677 |
| RS_RS19570 | - | hypothetical protein | -1.9361 | 1.0193 |
| RS_RS20360 | - | 5-methyltetrahydropteroyltriglutamate--homocysteine methyltransferase | -2.0234 | 3.1727 |
| RS_RS20430 | *fahA* | fumarylacetoacetase | -1.0902 | - |
| RS_RS20435 | - | homogentisate 1%2C2-dioxygenase | -1.099 | - |
| RS_RS20590 | - | radical SAM protein | -1.3758 | -2.8294 |
| RS_RS20835 | - | GNAT family N-acetyltransferase | -1.1261 | - |
| RS_RS20840 | - | alpha/beta hydrolase | -1.3716 | - |
| RS_RS20900 | - | acyl-CoA desaturase | -1.1015 | - |
| RS_RS21045 | - | GntR family transcriptional regulator | -1.727 | - |
| RS_RS21790 | - | U32 family peptidase | -1.7301 | -2.2838 |
| RS_RS21795 | - | U32 family peptidase | -2.4757 | -1.8947 |
| RS_RS21800 | - | hypothetical protein | -1.8079 | -1.5352 |
| RS_RS21840 | - | nitrate/nitrite sensor histidine kinase | -2.7805 | -1.5352 |
| RS_RS21845 | - | DNA-binding response regulator | -2.1716 | -2.0151 |
| RS_RS22545 | - | NAD(P)-dependent alcohol dehydrogenase | -1.2037 | - |
| RS_RS22870 | - | permease | -1.0697 | - |
| RS_RS23980 | - | non-ribosomal peptide synthetase | -1.0234 | - |
| RS_RS24010 | - | rhodanese-like domain-containing protein | -1.0404 | - |
| RS_RS24360 | *nirK* | nitrite reductase%2C copper-containing | -1.9754 | -1.8995 |
| RS_RS24625 | - | universal stress protein | 1.115 | -1.4159 |
| RS_RS24960 | - | NAD(P)-dependent oxidoreductase | -1.1762 | 1.5677 |
| RS_RS25085 | - | hypothetical protein | -1.4548 | - |
| RS_RS25920 | - | hypothetical protein | 2.7147 | - |
| RS_RS00015 | - | membrane protein insertase YidC | - | 1.485 |
| RS_RS00165 | *argB* | acetylglutamate kinase | - | 1.219 |
| RS_RS00210 | - | HslU--HslV peptidase ATPase subunit | - | 1.2601 |
| RS_RS00215 | - | HslU--HslV peptidase proteolytic subunit | - | 1.3844 |
| RS_RS00320 | - | AMP-dependent synthetase | - | -1.5825 |
| RS_RS00345 | - | LysM peptidoglycan-binding domain-containing protein | - | 1.2147 |
| RS_RS00405 | - | hypothetical protein | - | 1.0053 |
| RS_RS00685 | - | orotate phosphoribosyltransferase | - | 1.5826 |
| RS_RS00720 | - | YdcF family protein | - | 1.6222 |
| RS_RS00855 | - | membrane protein | - | 1.3088 |
| RS_RS00885 | - | universal stress protein | - | -2.1836 |
| RS_RS00900 | - | DUF2892 domain-containing protein | - | -3.0324 |
| RS_RS00910 | - | universal stress protein | - | -1.9707 |
| RS_RS00915 | - | universal stress protein | - | -2.7341 |
| RS_RS00920 | - | BON domain-containing protein | - | -2.8772 |
| RS_RS00925 | - | hypothetical protein | - | -2.5677 |
| RS_RS00960 | - | alcohol dehydrogenase | - | -2.1352 |
| RS_RS00965 | - | ribonucleoside-diphosphate reductase%2C adenosylcobalamin-dependent | - | -1.7782 |
| RS_RS00980 | - | hypothetical protein | - | -1.8109 |
| RS_RS00985 | - | cytochrome b | - | -1.6455 |
| RS_RS00990 | - | Hsp20/alpha crystallin family protein | - | -3.1712 |
| RS_RS00995 | - | MBL fold metallo-hydrolase | - | -3.3956 |
| RS_RS01000 | - | phosphoribosylpyrophosphate synthetase | - | -3.1327 |
| RS_RS01005 | - | DUF1488 domain-containing protein | - | -2.6247 |
| RS_RS01155 | - | DUF427 domain-containing protein | - | -1.1462 |
| RS_RS01375 | - | MerR family DNA-binding transcriptional regulator | - | -1.2137 |
| RS_RS01380 | - | Zn-dependent hydrolase | - | -1.1251 |
| RS_RS01575 | - | hypothetical protein | - | -2.7133 |
| RS_RS01625 | - | C4-dicarboxylate transporter 1 | - | 1.1822 |
| RS_RS01765 | *trmL* | tRNA(uridine(34)/cytosine(34)/5-carboxymethylaminomethyluridine(34)-2'-O)-methyltransferase TrmL | - | 1.35 |
| RS_RS02055 | - | aryl-sulfate sulfotransferase | - | -1.507 |
| RS_RS02060 | - | protease | - | -2.6845 |
| RS_RS02150 | - | acyl carrier protein | - | 1.0056 |
| RS_RS02215 | - | acetate kinase | - | -1.0232 |
| RS_RS02340 | - | TetR/AcrR family transcriptional regulator | - | -1.3068 |
| RS_RS02345 | - | acyl-CoA dehydrogenase | - | -1.0884 |
| RS_RS02380 | - | Glu/Leu/Phe/Val dehydrogenase | - | 1.3048 |
| RS_RS02385 | - | amino acid ABC transporter substrate-binding protein | - | 1.4391 |
| RS_RS02390 | - | amino acid ABC transporter permease | - | 1.4645 |
| RS_RS02395 | - | glutamate/aspartate transporter permease GltK | - | 1.4786 |
| RS_RS02400 | - | amino acid ABC transporter ATP-binding protein | - | 1.29 |
| RS_RS02490 | - | Holliday junction branch migration protein RuvA | - | 1.2379 |
| RS_RS02705 | - | pirin family protein | - | -1.437 |
| RS_RS02915 | - | avrd-related protein | - | 1.3763 |
| RS_RS02960 | - | hypothetical protein | - | -1.0526 |
| RS_RS02970 | - | hypothetical protein | - | -1.2063 |
| RS_RS04030 | - | membrane protein | - | -1.2793 |
| RS_RS04085 | - | LysE family translocator | - | -1.1971 |
| RS_RS04330 | - | type III secretion system YopJ family effector PopP2 | - | -1.6763 |
| RS_RS04340 | - | PIN domain-containing protein | - | -4.7943 |
| RS_RS04345 | - | type II toxin-antitoxin system Phd/YefM family antitoxin | - | -4.0468 |
| RS_RS04415 | - | hypothetical protein | - | 1.7423 |
| RS_RS04715 | - | serine/threonine dehydratase | - | -2.737 |
| RS_RS04725 | *egtD* | L-histidine N(alpha)-methyltransferase | - | -1.4857 |
| RS_RS04920 | - | membrane protein | - | 1.4774 |
| RS_RS04940 | - | MoxR family ATPase | - | -1.1812 |
| RS_RS04950 | - | cytochrome c | - | 1.1434 |
| RS_RS05130 | - | hypothetical protein | - | 1.6552 |
| RS_RS05215 | - | acyl carrier protein | - | 1.0333 |
| RS_RS05515 | - | LysR family transcriptional regulator | - | -1.5006 |
| RS_RS05675 | - | PHP domain-containing protein | - | -1.005 |
| RS_RS05680 | - | threonylcarbamoyl-AMP synthase | - | -1.2457 |
| RS_RS05845 | - | enoyl-[acyl-carrier-protein] reductase FabI | - | 1.3502 |
| RS_RS06335 | - | sn-glycerol-3-phosphate ABC transporter substrate-binding protein UgpB | - | 1.1461 |
| RS_RS06350 | - | sn-glycerol-3-phosphate import ATP-binding protein UgpC | - | 1.1834 |
| RS_RS06380 | - | 2-phosphosulfolactate phosphatase | - | -1.1962 |
| RS_RS06390 | *ccoS* | cbb3-type cytochrome oxidase assembly protein CcoS | - | -1.485 |
| RS_RS06480 | - | hemolysin D | - | -1.019 |
| RS_RS06485 | - | outer membrane CHANEL lipoprotein | - | -2.6383 |
| RS_RS06490 | - | MarR family transcriptional regulator | - | -2.5697 |
| RS_RS06550 | - | asparaginase | - | 1.074 |
| RS_RS06600 | - | PhoH family protein | - | -1.5576 |
| RS_RS06760 | - | CysB family HTH-type transcriptional regulator | - | -1.4962 |
| RS_RS06765 | - | type III effector protein | - | -1.0815 |
| RS_RS06785 | - | thiamine pyrophosphate protein | - | -1.3659 |
| RS_RS06790 | - | ribonuclease E inhibitor RraA | - | -1.4369 |
| RS_RS06795 | - | gamma-glutamylcyclotransferase | - | -1.5485 |
| RS_RS07360 | - | hypothetical protein | - | -1.5308 |
| RS_RS07365 | - | hypothetical protein | - | -1.0367 |
| RS_RS07450 | - | hypothetical protein | - | -2.1134 |
| RS_RS07515 | - | hypothetical protein | - | -1.5001 |
| RS_RS07520 | - | hypothetical protein | - | -2.3495 |
| RS_RS07525 | - | hypothetical protein | - | -1.9821 |
| RS_RS07565 | - | zinc-binding alcohol dehydrogenase family protein | - | -1.595 |
| RS_RS07570 | - | DUF2892 domain-containing protein | - | -2.088 |
| RS_RS07575 | - | hypothetical protein | - | -1.547 |
| RS_RS08140 | - | leucyl/phenylalanyl-tRNA--protein transferase | - | -1.0526 |
| RS_RS08165 | - | hypothetical protein | - | -1.3463 |
| RS_RS08630 | - | hypothetical protein | - | -1.3744 |
| RS_RS08635 | - | bifunctional tRNA (5-methylaminomethyl-2-thiouridine)(34)-methyltransferase MnmD/FAD-dependent 5-carboxymethylaminomethyl-2-thiouridine(34) oxidoreductase MnmC | - | -1.0831 |
| RS_RS08720 | - | DUF1842 domain-containing protein | - | -1.5432 |
| RS_RS08725 | - | DUF1842 domain-containing protein | - | -1.0251 |
| RS_RS08730 | - | GDL motif peptide-associated radical SAM/SPASM maturase | - | -1.1419 |
| RS_RS08810 | - | peptidyl-prolyl cis-trans isomerase | - | 1.3318 |
| RS_RS08830 | - | YdiU family protein | - | -1.3131 |
| RS_RS08835 | - | long-chain-fatty-acid--CoA ligase | - | 1.0131 |
| RS_RS08870 | - | exo-poly-galacturonase signal peptide protein | - | 1.1401 |
| RS_RS09170 | - | methionine--tRNA ligase | - | -1.0057 |
| RS_RS09175 | - | cupin domain-containing protein | - | -1.2093 |
| RS_RS09330 | - | hypothetical protein | - | -1.4165 |
| RS_RS09370 | - | DUF1794 domain-containing protein | - | -1.1266 |
| RS_RS09555 | - | DNA-binding response regulator | - | 1.1024 |
| RS_RS09840 | - | dicarboxylate/amino acid:cation symporter | - | 1.1488 |
| RS_RS09915 | - | glycine zipper 2TM domain-containing protein | - | 1.4843 |
| RS_RS09960 | - | site-specific DNA-methyltransferase | - | -1.2752 |
| RS_RS09965 | *trpB* | tryptophan synthase subunit beta | - | -3.6098 |
| RS_RS09970 | - | phosphoribosylanthranilate isomerase | - | -2.5235 |
| RS_RS10510 | *xdhA* | xanthine dehydrogenase small subunit | - | -1.2984 |
| RS_RS10515 | *xdhB* | xanthine dehydrogenase molybdopterin binding subunit | - | -1.4314 |
| RS_RS10570 | - | hypothetical protein | - | -1.167 |
| RS_RS10855 | - | DUF2383 domain-containing protein | - | -1.1769 |
| RS_RS10875 | - | DUF979 domain-containing protein | - | 1.544 |
| RS_RS10880 | - | DUF969 domain-containing protein | - | 1.6437 |
| RS_RS10885 | - | LamB/YcsF family protein | - | 1.4031 |
| RS_RS10890 | - | allophanate hydrolase | - | 1.3822 |
| RS_RS10895 | - | hypothetical protein | - | 1.8825 |
| RS_RS11245 | - | hypothetical protein | - | -1.4028 |
| RS_RS11255 | *pobA* | 4-hydroxybenzoate 3-monooxygenase | - | -2.1808 |
| RS_RS11615 | - | hypothetical protein | - | -1.0831 |
| RS_RS11795 | - | inorganic pyrophosphatase | - | 1.3121 |
| RS_RS11815 | - | hypothetical protein | - | 1.5337 |
| RS_RS11820 | - | cytochrome B oxidoreductase | - | 1.0489 |
| RS_RS12155 | - | sulfate adenylyltransferase subunit CysD | - | -1.0024 |
| RS_RS12235 | - | ABC transporter ATP-binding protein | - | 1.1594 |
| RS_RS12240 | - | ABC transporter ATP-binding protein | - | 1.1262 |
| RS_RS12245 | - | ABC transporter ATP-binding protein | - | 1.3618 |
| RS_RS12250 | - | branched-chain amino acid ABC transporter permease | - | 1.6075 |
| RS_RS12255 | - | branched-chain amino acid ABC transporter substrate-binding protein | - | 1.7759 |
| RS_RS12535 | - | DoxX family protein | - | -1.3689 |
| RS_RS12675 | - | MotA/TolQ/ExbB proton channel family protein | - | 1.4494 |
| RS_RS12685 | - | tetraacyldisaccharide 4'-kinase | - | 1.2664 |
| RS_RS12705 | - | 3-hydroxyacyl-CoA dehydrogenase | - | -1.8173 |
| RS_RS12745 | - | membrane protein | - | 2.4914 |
| RS_RS12830 | - | membrane protein | - | 1.5175 |
| RS_RS12975 | - | D-alanyl-D-alanine endopeptidase | - | -1.0656 |
| RS_RS13260 | *hutC* | histidine utilization repressor | - | 1.3392 |
| RS_RS13385 | - | YafY family transcriptional regulator | - | 1.313 |
| RS_RS13510 | - | hypothetical protein | - | -1.3768 |
| RS_RS13655 | - | TonB-dependent siderophore receptor | - | 1.3568 |
| RS_RS13875 | *corA* | magnesium and cobalt transport protein CorA | - | 1.2775 |
| RS_RS13930 | *aroQ* | 3-dehydroquinate dehydratase 1 | - | 1.3877 |
| RS_RS13935 | *accB* | acetyl-CoA carboxylase biotin carboxyl carrier protein | - | 1.0787 |
| RS_RS14325 | - | ABC transporter permease | - | 1.2145 |
| RS_RS14465 | - | DUF2778 domain-containing protein | - | 1.3851 |
| RS_RS14470 | - | hypothetical protein | - | 1.0168 |
| RS_RS14610 | *cyoA* | ubiquinol oxidase subunit II | - | 1.3513 |
| RS_RS14725 | - | ABC transporter ATP-binding protein | - | 1.2663 |
| RS_RS14865 | - | transposase | - | -1.0565 |
| RS_RS15200 | *rpoB* | DNA-directed RNA polymerase subunit beta | - | -1.1144 |
| RS_RS15290 | - | CHAD domain-containing protein | - | -1.2848 |
| RS_RS15295 | - | DeoR/GlpR transcriptional regulator | - | -2.4869 |
| RS_RS15300 | - | glycerol-3-phosphate dehydrogenase | - | -1.2605 |
| RS_RS15560 | - | N-acetyltransferase | - | -1.1438 |
| RS_RS15575 | - | serine protease | - | 1.4402 |
| RS_RS15725 | - | alcohol dehydrogenase AdhP | - | -1.1936 |
| RS_RS15730 | - | 2-hydroxyacid dehydrogenase | - | -1.2136 |
| RS_RS15825 | - | hypothetical protein | - | 1.3784 |
| RS_RS16530 | - | potassium transporter Kef | - | 1.0281 |
| RS_RS16535 | - | branched-chain amino acid ABC transporter substrate-binding protein | - | 1.4937 |
| RS_RS16540 | *putA* | trifunctional transcriptional regulator/proline dehydrogenase/L-glutamate gamma-semialdehyde dehydrogenase | - | 1.6489 |
| RS_RS16650 | - | F0F1 ATP synthase subunit A | - | 1.1061 |
| RS_RS16680 | - | branched-chain amino acid ABC transporter substrate-binding protein | - | 1.694 |
| RS_RS16735 | - | branched-chain amino acid ABC transporter permease | - | 1.097 |
| RS_RS16740 | - | branched-chain amino acid ABC transporter permease | - | 1.8907 |
| RS_RS16745 | - | ABC transporter permease | - | 1.9155 |
| RS_RS16755 | - | ABC transporter ATP-binding protein | - | 2.324 |
| RS_RS16915 | - | hypothetical protein | - | -1.7628 |
| RS_RS16920 | - | hypothetical protein | - | -1.6843 |
| RS_RS16925 | - | hypothetical protein | - | -1.4783 |
| RS_RS17205 | - | cold-shock protein | - | 1.2085 |
| RS_RS17260 | - | amino acid ABC transporter substrate-binding protein | - | 1.3644 |
| RS_RS17265 | - | branched-chain amino acid ABC transporter permease | - | 1.5705 |
| RS_RS17270 | - | branched-chain amino acid ABC transporter permease | - | 1.3673 |
| RS_RS17765 | - | DNA helicase II | - | -1.1635 |
| RS_RS17770 | - | TIR domain-containing protein | - | -1.1242 |
| RS_RS17790 | - | FUSC family protein | - | -2.6129 |
| RS_RS18095 | - | gamma subclass chorismate mutase AroQ | - | 1.4296 |
| RS_RS18155 | - | phytanoyl-CoA dioxygenase | - | -1.089 |
| RS_RS18515 | - | APC family permease | - | 1.642 |
| RS_RS18575 | - | calcium-binding protein | - | -1.0163 |
| RS_RS18665 | - | nitronate monooxygenase | - | -1.0902 |
| RS_RS18670 | - | nuclear transport factor 2 family protein | - | -2.052 |
| RS_RS18675 | - | universal stress protein | - | -1.4044 |
| RS_RS18750 | - | hypothetical protein | - | 1.2118 |
| RS_RS18775 | - | DNA-binding response regulator | - | -1.5155 |
| RS_RS19240 | - | hypothetical protein | - | -1.9037 |
| RS_RS19245 | - | hypothetical protein | - | -1.8173 |
| RS_RS19530 | - | CusA/CzcA family heavy metal efflux RND transporter | - | 1.215 |
| RS_RS19655 | - | GGDEF domain-containing protein | - | 2.0271 |
| RS_RS19740 | - | MFS transporter | - | 2.2448 |
| RS_RS19745 | - | hypothetical protein | - | 1.9815 |
| RS_RS19995 | - | hypothetical protein | - | 1.1317 |
| RS_RS20005 | - | 1%2C2-phenylacetyl-CoA epoxidase subunit A | - | -1.6359 |
| RS_RS20300 | - | transcriptional regulator | - | 1.2238 |
| RS_RS20370 | *pdxH* | pyridoxamine 5'-phosphate oxidase | - | -2.1477 |
| RS_RS20375 | - | diaminopimelate decarboxylase | - | -2.3825 |
| RS_RS20390 | - | NADH:flavin oxidoreductase | - | -1.0831 |
| RS_RS20595 | - | hypothetical protein | - | -1.578 |
| RS_RS20995 | - | TonB-dependent receptor | - | 1.4988 |
| RS_RS21205 | - | DNA-binding response regulator | - | 1.3698 |
| RS_RS21740 | - | nitric oxide reductase transcriptional regulator NorR | - | -1.5132 |
| RS_RS21760 | - | ribonucleoside triphosphate reductase | - | -1.9123 |
| RS_RS21775 | - | Crp/Fnr family transcriptional regulator | - | -2.5796 |
| RS_RS21780 | - | zinc-binding protein | - | -2.2902 |
| RS_RS21785 | - | SCP2 domain-containing protein | - | -2.3173 |
| RS_RS21870 | - | MFS transporter | - | 1.126 |
| RS_RS21875 | - | 4-hydroxybutyrate dehydrogenase | - | 1.0033 |
| RS_RS21930 | - | tartrate dehydrogenase | - | -1.721 |
| RS_RS22040 | - | protein-tyrosine-phosphatase | - | 1.0436 |
| RS_RS22305 | - | ShlB/FhaC/HecB family hemolysin secretion/activation protein | - | 1.3673 |
| RS_RS22420 | - | endonuclease | - | 1.3984 |
| RS_RS22455 | - | MarR family transcriptional regulator | - | 1.0646 |
| RS_RS22465 | - | alpha/beta hydrolase | - | 1.3743 |
| RS_RS22760 | - | choline dehydrogenase | - | -1.2326 |
| RS_RS22965 | - | GGDEF domain-containing protein | - | -1.1554 |
| RS_RS23335 | *ptsP* | phosphoenolpyruvate--protein phosphotransferase | - | -2.3663 |
| RS_RS23340 | - | maltoporin | - | -2.5469 |
| RS_RS23345 | - | fructosidase | - | -2.588 |
| RS_RS23350 | - | PTS sugar transporter | - | -2.1513 |
| RS_RS23705 | - | acyltransferase | - | -1.1481 |
| RS_RS23720 | - | TAT-dependent nitrous-oxide reductase | - | -1.819 |
| RS_RS23760 | - | branched-chain amino acid ABC transporter permease | - | 2.0313 |
| RS_RS23825 | - | MBL fold metallo-hydrolase | - | -1.158 |
| RS_RS23960 | - | MFS transporter | - | 1.2192 |
| RS_RS23970 | - | peptide synthetase | - | 1.0992 |
| RS_RS24065 | - | serine acetyltransferase | - | 1.1743 |
| RS_RS24350 | - | SCO family protein | - | -2.2929 |
| RS_RS24355 | - | formylglycine-generating enzyme family protein | - | -2.4214 |
| RS_RS24475 | - | rhomboid family intramembrane serine protease | - | 1.1586 |
| RS_RS24480 | - | DUF3500 domain-containing protein | - | 1.3972 |
| RS_RS24635 | - | hypothetical protein | - | -1.2703 |
| RS_RS24640 | - | cytochrome B558 subunit A | - | -1.0441 |
| RS_RS24725 | - | catalase | - | 1.4344 |
| RS_RS24940 | - | porin | - | 1.8376 |
| RS_RS24945 | *xylF* | D-xylose ABC transporter substrate-binding protein | - | 1.7905 |
| RS_RS24950 | - | D-xylose ABC transporter ATP-binding protein | - | 1.2935 |
| RS_RS24955 | - | sugar ABC transporter permease | - | 1.1882 |
| RS_RS24970 | - | EamA/RhaT family transporter | - | 2.0429 |
| RS_RS25045 | - | hypothetical protein | - | -1.4181 |
| RS_RS25220 | *rpmH* | 50S ribosomal protein L34 | - | 1.098 |
| RS_RS25270 | - | hypothetical protein | - | -1.0446 |
| RS_RS25535 | *ssrS* | 6S RNA | - | -1.0202 |
| RS_RS25760 | - | hypothetical protein | - | -1.443 |
| RS_RS25765 | - | hypothetical protein | - | -1.9343 |
| RS_RS26005 | - | serine/threonine protein kinase | - | -1.8792 |
| RS_RS26200 | - | MbtH family protein | - | 1.2589 |
| RS_RS26570 | - | hypothetical protein | - | 1.2115 |
