# Supplementary material for: Ralstonia solanacearum promotes pathogenicity by utilizing l‐glutamic acid from host plants
Source: Mol Plant Pathol. 2020 Jun 29;21(8):1099–110. doi: 10.1111/mpp.12963 (PMC7368120; doi:10.1111/mpp.12963)
Supplement: Supplementary file 9 — TABLE S2 PCR primers used in this study [file MPP-21-1099-s009.docx]

**Table S2.** PCR primers used in this study.

| **Primer** | **Sequence (5’-3’)** |
| --- | --- |
| **For report strain** |  |
| *epsA*-F | CCCAAGCTTCACGGGCTGCAAGCTCCTG |
| *epsA*-R | CCGCTCGAGCACGCTCAACGACACGACTG |
| **For gene knockout** |  |
| RS01577L-F | CCGGAATTCCTGAACGCAGCCATCCAGGCCG |
| RS01577L-R | CGTCGCCCGTTCAGCCCGGATGCATGAGGGCT |
| RS01577R-F | CTTTTTGGAAGCCCTCATGCATCCGGGCTGAAC |
| RS01577R-R | CCGCGGATCCTGGGATTGCAAGATGCG |
| pK18-F | CAATTAATGTGAGTTAGCTCACTC |
| pK18-R | CATTCAGGCTGCGCAACTGTTGG |
| **For qPCR analysis** |  |
| REC-F | GGCAAGACCACGCTGACGCT |
| REC-R | TTTCCAGCGCCTGCTCACCG |
| RS05516-F | CGGTGTCGCCAAGGTATG |
| RS05516-R | GCGGACGGATAGATGTAGTTG |
| EpsA-F | AAAGCAGTCGTGTCGTTGAG |
| EpsA-R | GCGTAATGGGCGTGATCTT |
| EpsE-F | GCAAGTTCTGGCGCAATTT |
| EpsE-R | GAACAGGATCAGGCAGCAATA |
| EpsF-F | TGCGTTCTACGAGTTCCAGC |
| EpsF-R | TTGGCCACGGAATACGAGAG |
| PheC-F | AGCAGGACCAGAACTACCT |
| PheC-R | TTGGTGCAGCCGATGAAT |
| MotA-F | GCTAGTCGCCATCGGTTACA |
| MotA-R | GATCGCCTTCTTGTCGTTGC |
| PilP-F | GGTAACGCGTCTCATACATCTT |
| PilP-R | TGCCTGCTGCATCCATT |
| RS_RS00055-F | GCGGAAGAGGTGTTCTATGT |
| RS_RS00055-R | TGCGGAAATGCCAGTAGAT |
| RS_RS00905-F | ATGAGGGTCAGCGAGATTTG |
| RS_RS00905-R | ACATTGGTCCCGCATCA |
| RS_RS01070-F | ATGATGTCCACGCTGATGAC |
| RS_RS01070-R | GACCACAGCTTCAGGATCAG |
| RS_RS01075-F | AGGCAAGGTCAGGCATTT |
| RS_RS01075-R | GCGTCCACAGCGAATACT |
| RS_RS03840-F | CAAGAAATTTCCACTGCATCCC |
| RS_RS03840-R | CATTCGTACCAGTCGTCACC |
| RS_RS06385-F | CGCCAGTCCCGAAGATAAG |
| RS_RS06385-R | AGCGGTGCATCGTTGAT |
| RS_RS06725-F | CATCGACATGCAGAACCTGA |
| RS_RS06725-R | CACATCGTGGGTGACCAG |
| RS_RS06915-F | CCTGCAGAAGGAATTCGGTATC |
| RS_RS06915-R | ATCTGGCCCAGGTACATCA |
| RS_RS06920-F | GAACACCGCGTACTACAAGAA |
| RS_RS06920-R | GGTGTACAGCTTGGTCTTCTC |
| RS_RS06925-F | GTGGTGGAGAAGGTGTTCAA |
| RS_RS06925-R | CAAGATGAACTCCAGCGAGAA |
| RS_RS06930-F | AAGCAGCATCTGGCACTC |
| RS_RS06930-R | AGCGCGTCATAGTCGAAATAG |
| RS_RS06935-F | CTGGTGCTGGCCAACTT |
| RS_RS06935-R | CATGATGATGGAGCCCTTCT |
| RS_RS14900-F | TGCTTGGCTGGCAGTTT |
| RS_RS14900-R | CTTGGACTGGTATGCGGATTT |
| RS_RS16945-F | CATCCTGGCGGTGTTCAT |
| RS_RS16945-R | GGAGGTCATCTTCATCTCGAAC |
| RS_RS18995-F | ACCAACCTGGACAAGGAATAC |
| RS_RS18995-R | GCTTGTTGCCGTTGTAGTTG |
| RS_RS00320-F | ATCCTGCTGTATCGCTTCG |
| RS_RS00320-R | CCATCACCGCCAGATTCAT |
| RS_RS16455-F | CGCAACTTCCAGGGCTATT |
| RS_RS16455-R | TCGTTCTGCGCATAGACATC |
| RS_RS20590-F | GCAGTGTCCGGTGGATTATTA |
| RS_RS20590-R | CGGAGAACGCATCGGTAAA |
| RS_RS21840-F | AAGCAACGCCCACAAGA |
| RS_RS21840-R | TCCACAGGTACAGCAGATAGA |
| RS_RS00995-F | CATCCTGATGTCAGGGTTTCA |
| RS_RS00995-R | TGGCACGAATCGCAATCT |
| RS_RS24360-F | AGGTGAATGGCCAGGAATAC |
| RS_RS24360-R | GAGCACATAGGTCAGGATGTT |
| RS_RS24625-F | AGGTGAATGGCCAGGAATAC |
| RS_RS24625-R | GAGCACATAGGTCAGGATGTT |
| RS_RS04330-F | TACGCCGACAACGTGAATATG |
| RS_RS04330-R | GAGTATCCGGCAATCGAAGAAG |
| RS_RS04345-F | TTTCCGATGTCGTGAAGCG |
| RS_RS04345-R | CATGAAGCTGACCAGTGACTC |
| RS_RS00910-F | GTGATTGACAACGCCTACCT |
| RS_RS00910-R | CATCGGTGACGATCCGTTT |
| RS_RS02385-F | GGCTCCACCACCAATAATCT |
| RS_RS02385-R | CGGAATCCTTCTTCACCATCA |
| RS_RS20360-F | TTCGGAGTTCAACGACATCC |
| RS_RS20360-R | TCCAGCAGCTCCATGTT |
| RS_RS00685-F | AGTTGCGCCAATCCTTCAT |
| RS_RS00685-R | TGCCGGCGTTGAAGAAATA |
| RS_RS00720-F | CTCGAACCCAACAGCATGAA |
| RS_RS00720-R | GCCGAAGTGCGCGAAATA |

* Restriction enzyme site is underlined.
